# Volumetric Absorptive Microsampling in the Analysis of Endogenous Metabolites

**DOI:** 10.3390/metabo13101038

**Published:** 2023-09-26

**Authors:** Daniel Marques de Sá e Silva, Marlene Thaitumu, Georgios Theodoridis, Michael Witting, Helen Gika

**Affiliations:** 1 Department of Chemistry, Aristotle University of Thessaloniki (AUTH), 54124 Thessaloniki, Greecegtheodor@chem.auth.gr (G.T.); 2 Biomic_AUTh, Center for Interdisciplinary Research and Innovation (CIRI-AUTH), Balkan Center, B1.4, 57001 Thessaloniki, Greece; marlenet96@gmail.com; 3 School of Medicine, Aristotle University of Thessaloniki, 54124 Thessaloniki, Greece; 4 Metabolomics and Proteomics Core, Helmholtz Zentrum München, Ingolstädter Landstraße 1, 85764 Neuherberg, Germany; 5 Chair of Analytical Food Chemistry, TUM School of Life Sciences, Technical University of Munich, Maximus-von-Imhof Forum 6, 85354 Freising, Germany

**Keywords:** volumetric absorptive microsampling (VAMS), blood microsampling, endogenous metabolites, biomarkers, analytical validation, clinical application

## Abstract

Volumetric absorptive microsampling (VAMS) has arisen as a relevant tool in biological analysis, offering simplified sampling procedures and enhanced stability. Most of the attention VAMS has received in the past decade has been from pharmaceutical research, with most of the published work employing VAMS targeting drugs or other exogenous compounds, such as toxins and pollutants. However, biomarker analysis by employing blood microsampling has high promise. Herein, a comprehensive review on the applicability of VAMS devices for the analysis of endogenous metabolites/biomarkers was performed. The study presents a full overview of the analysis process, incorporating all the steps in sample treatment and validation parameters. Overall, VAMS devices have proven to be reliable tools for the analysis of endogenous analytes with biological importance, often offering improved analyte stability in comparison with blood under ambient conditions as well as a convenient and straightforward sample acquisition model.

## 1. Introduction

A key aim of analytical scientists has always been to improve the accuracy, efficiency, and cost effectiveness of quantitative bioanalytical assays. This goal is pursued through various strategies, including automation techniques and the miniaturization of sample collection, preparation, and analysis. The miniaturization of collection (microsampling) is arguably an important aspect, as it affects every downstream process and technology. Additionally, modern liquid chromatography–tandem mass spectrometry (LC-MS/MS) instrumentation is now highly advanced, sensitive, and automated, making it easy to integrate microsamples and their extraction strategies and consequently leading to higher efficiency, lower costs, and higher throughput [1,2,3].

Microsampling involves the collection of very small volumes (µL) from a matrix. The earliest known form of microsampling for bioanalysis is dried blood spot (DBS). Its first use was reported in 1916 and involved the analysis of glucose on a sample collected on filter paper. In 1961, DBS analysis was used for phenylketonuria (PKU) assessment via the analysis of phenylalanine [3,4]; since then, DBS analysis has been the standard procedure for newborn screening.

DBS analysis provides numerous advantages over standard venipuncture for blood collection. Collection is minimally invasive and simple, thus allowing remote and home collection; there is no need for trained staff at hospitals and no or only a minimal need for controlled transport temperature conditions. Furthermore, it is non-intrusive for phlebotomy-reluctant populations (newborns, children, the elderly, and generally vulnerable people) and extraction and analysis can be automated in assays such as LC-MS/MS. Lastly, it has helped to improve the welfare of animals and to reduce the number of required specimens for scientific studies [2,3,5,6].

Despite the numerous advantages, DBS analysis presents challenging issues, the main one being the hematocrit (HCT) effect. Variability in HCT among individuals leads to variations in blood viscosity, which affects the diffusion of blood on the paper of the DBS cards. This leads to different blood spot sizes and, consequently, compromised analytical results. Additionally, the chromatographic effect that takes place when applying the blood drop to the DBS card leads to non-homogeneity of analytes in the blood spots, which may also compromise results [3,6,7]. For these reasons, the scientific community has opted for other microsampling techniques that combine the advantages of DBS analysis while mitigating its challenges.

Volumetric absorptive microsampling (VAMS) is a technique developed to mitigate issues faced in DBS analysis. VAMS allows the absorption of a very small fixed volume of blood onto a porous polymeric tip, which is later dried.

Since the conception and commercialization of this principle in 2014 [6], there have been approximately 100 articles published reporting analytical assays using VAMS; the overwhelming majority have been on pharmacokinetic, toxicologic, and therapeutic drug monitoring applications [2,3,5,6]. Very recently, a review article by Nugraha et al. was published that focused on studies in which optimization and validation assays employing VAMS in the analysis of pharmaceuticals were performed [8] and provided details on the analytical protocols and their clinical validation in the framework of VAMS application in drug analysis.

Nonetheless, the utilization of VAMS in the analysis of endogenous metabolites, including proteins, has emerged very recently, aiming towards either disease diagnosis and monitoring, nutritional deficiency assessments, drug monitoring, alcohol abuse, sports doping, or metabolomics research. Undoubtedly, accurate determination of endogenous metabolites/small molecule “biomarkers” in these fields could be greatly facilitated by such microsampling devices, opening new routes in disease diagnosis and health monitoring.

Endogenous metabolite analysis using VAMS is still in its early stages, with studies only beginning to be published in the last five years; therefore, research and validation studies still need to be conducted in order to further improve the use of VAMS for such analytes, mitigate its bottlenecks, and further optimize its general use, shipping, and storage, as well as extraction procedures. To achieve this, an extensive compilation of previous and current research is needed by other scientists in order to help them meet these goals. As such, this review will report on the analysis of various endogenous metabolites/small molecule biomarkers using VAMS. A focus is given to the sensitivity, selectivity, accuracy, precision, stability, matrix effects, and robustness of these methodologies as well as the controversial issue of HCT effects. Furthermore, we provide insights into clinical studies and the advantages obtained with VAMs over blood by comparing the results obtained by VAMS sampling methods (capillary or venous blood) with traditional sampling techniques (whole blood and plasma). The reported applications include metabolites such as vitamins, an ethanol metabolite (phosphatidylethanol), anabolic androgenic metabolites, creatinine, amino, aromatic, organic, and carboxylic acids, and their derivatives. The collection of articles studied is summarized in Table 1. The aim is to extensively cover the current state-of-the-art VAMS applications for endogenous metabolites as a starting point for scientists aiming to use VAMS for such bioanalytical purposes.

## 2. VAMS Technology

**Figure 1:**
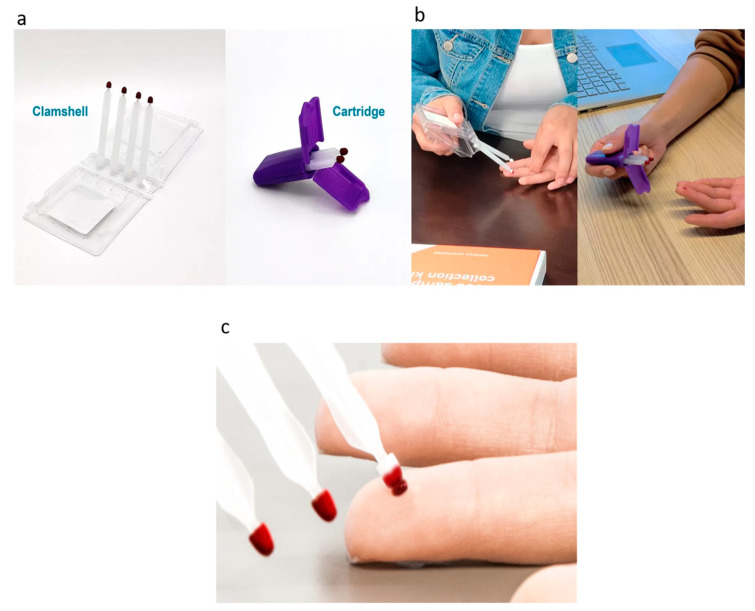
VAMS procedure: (**a**) VAMS tips are attached to a clamshell or cartridge; (**b**) tips are held at an angle to the blood, then removed after they are completely filled; [9] (**c**) closeup of the blood absorption [3]. Copyright (2023), with permission from Elsevier.

The commercial VAMS technology that has been introduced into the market uses a device that includes an adsorbent hydrophilic polymeric probe attached to a closable clamshell or cartridge (Figure 1a) [2]. The tips are designed to draw exact volumes (e.g., 10, 20, or 30 µL) of blood and therefore limit variations and inconsistent sampling [3,6]. For collection, the polymeric tip is placed at an angle to the sample (capillary blood after a finger or heel prick and venous blood) surface where it draws the matrix of choice using capillary action until it is filled (Figure 1c) [6]. For best results, care needs to be taken not to overfill (by submerging the tip), drop, or rub the tips against other surfaces. After drawing a sample, the tips are air dried before being sent for analysis in a vendor-provided bag containing desiccant [3,6].

**Figure 2:**
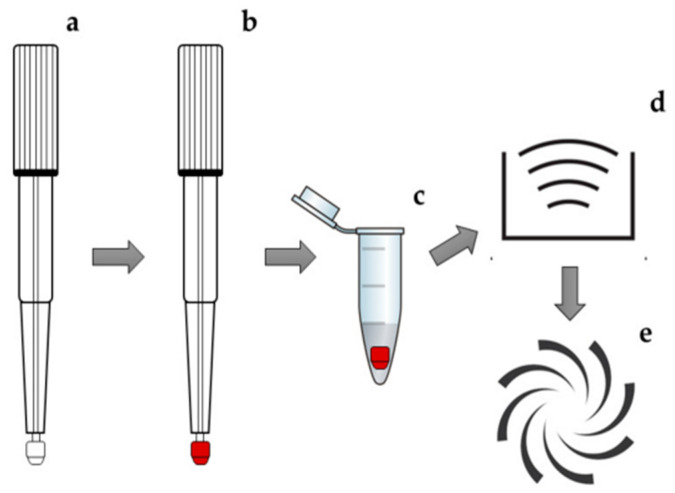
Representation of VAMS workflow: (**a**) unused VAMS sampler; (**b**) VAMS sampler after collection of blood; (**c**) VAMS tip extracted in an extraction solvent under; (**d**) ultrasound-assisted extraction (UAE); (**e**) vortex-assisted extraction (VAE). [10].

During sample extraction, the whole tip is usually detached from the cartridge or clamshell and placed in a microtube where the extraction solvent is added. Thereafter, vortexing or sonication is typically applied. A schematic presentation of the sample treatment can be seen in Figure 2. The solvents reportedly used for extraction include pure methanol, water, acetonitrile, or a percentage of the organic solvents in water. Depending on the analyte, pH adjustment is performed with (0.01–1%) formic acid. Nitric acid, trifluoroacetic acid, and 1% ammonia have also been used for the purpose of adjusting the pH [2].

For sample analysis, multiple techniques are available for consideration depending on the target and matrices. Since VAMS uses very small volumes, LC-MS/MS or GC-MS/MS are mostly used for different target analyte analyses, given their strengths in sensitivity, specificity, robustness, and throughput. Enzyme-linked immunosorbent assay (ELISA) has also been used for metabolomics studies [2,3].

As mentioned before, VAMS addresses DBS analysis issues while preserving its bioanalytical advantages. Deniff et al. reported no HCT effects on 10 µL VAMS blood samples that had HCTs ranging from 20 to 70% [11]. However, HCT effects have been observed in multiple pharmacokinetic studies, with low accuracies with high HCT and high accuracies with low HCT [3,5,12,13,14,15,16,17]. Since VAMS technology enables exact volume collection while ensuring homogeneity, the HCT effects may either be attributed to signal suppression/enhancement or varying recoveries for different HCTs. Parker et al. analyzed the drug fosfomycin and confirmed that signal suppression/enhancement was not an issue; other factors were causing varying recoveries at different HCTs [15]. One possible explanation was that high concentrations of red blood cells clog the pores in the VAMS tips and therefore limit extraction [14,15]. Thus, claims regarding the complete elimination of sampling inconsistencies when using VAMS, at least in pharmacokinetic studies, may not be completely accurate. Suggestions to mitigate HCT effects include optimization and careful selection of the extraction solvent [18], mixing times [15], and techniques (e.g., sonication) [13,14,17,19,20] during sample extraction in order to maximize recovery [3,5].

## 3. Method

In this review, publications that report the application of VAMS for the targeted analysis of endogenous metabolites in blood or urine are included. The literature search tools PubMed and Google Scholar were used by two independent researchers (authors DM and MT) based on the keywords: volumetric absorptive microsampling or Mitra microsampling; 217 articles were found. Five articles were considered irrelevant because they did not address VAMS. From the relevant articles, five were reviews, 163 were on drug analysis, 19 were on small molecule metabolites, 9 were on proteins and peptides, 5 were on metals, 5 were on COVID-19 diagnosis, 3 were on antibodies, and 2 were on mycotoxins and perfluorinated compounds. Publications on protein and peptide analytes, metals, COVID-19, pharmaceuticals, and nutrimedicals/exogenous compounds were excluded, as well as those on untargeted profiling/qualitative analysis of metabolites or metabolomics applications of VAMs. An overview of the applications studied herein is given in Table 1.

**Table 1:** Endogenous metabolites determined via VAMS.

Metabolite (+Secondary Analyte)	Specimen Sampled via VAMs	Analytical Technique	Analytical Validation	Clinical Validation Study	Reference
Creatinine (+vancomycin)	Plasma	LC-MS/MS	Table S1	Comparison of plasma VAMS vs. plasma fluid; 46 patients.	[21]
Creatinine (+tacrolimus)	Capillary finger blood	LC-MS/MS; Jaffe kinetic method		Comparison of capillary VAMS vs. DBS vs. serum fluid; 152 patients.	[22]
Creatinine (+tacrolimus)	Capillary finger blood	LC-MS/MS		Comparison of capillary VAMS vs. serum fluid; 135 patients.	[23]
Creatinine (+tacrolimus, mycophenolic acid, iohexol)	Capillary finger blood	LC-MS/MS		Comparison of capillary VAMS vs. DBS vs. serum fluid; 25 patients.	[24]
Creatinine (+tacrolimus, mycophenolic acid)	Capillary finger blood	LC-MS/MS; EMIT; COM		Comparison of capillary VAMS vs. DBS vs. serum fluid; 40 patients.	[25]
Creatinine (+tacrolimus)	Capillary finger blood	LC-MS/MS		Comparison of capillary VAMS vs. serum fluid; 40 patients.	[26]
Testosterone	Venous whole and capillary blood	GC-MS/MS	Table S2	Comparison of two GC-MS/MS systems; venous whole blood VAMS vs. serum fluid. Feasibility conducted on capillary VAMS (testosterone gel study administration); 40 patients.	[27]
Testosterone, Epitestosteorne, Dihydrotestoterone, Dehydroepiandrosterone	Urine	LC-MS/MS		Comparison of urine VAMS vs. dried urine spots (DUS) vs. fluid urine.	[28]
Cortisol, Cortisone, Corticosterone, 11-Dehydrocorticosterone	Venous whole blood (human and rats)	LC-MS/MS		Comparison of Mitra VAMS vs. Whatman 903 Protein Saver Card segment (DBS) vs. Noviplex Plasma Prep Card disc (dried plasma spot: DPS) vs. EDTA plasma fluid; 5 human and 8 rats.	[29]
Testosterone, Androstenedione, 17-Hydroxyprogesterone	Venous whole and capillary finger blood	LC-MS/MS		Comparison of venous whole blood vs. plasma; plasma vs. venous whole blood VAMS; serum vs. capillary blood VAMS; samples were from healthy volunteers.	[30]
25-Hydroxyvitamin D2 and D3	Venous whole and capillary finger blood	LC-MS/MS	Table S3	Optimization of venous whole blood VAMS using fortified samples; feasibility conducted on capillary VAMS from 20 healthy adults.	[31]
Thiamine diphosphate	Venous whole blood	LC-MS/MS		Comparison of venous whole blood VAMS vs. venous whole blood fluid at normal and extreme HCT levels; healthy human volunteers.	[32]
Thiamine diphosphate	Venous whole and capillary finger blood	LC-MS/MS		Comparison of venous whole blood VAMS vs. venous whole blood fluid; capillary VAMS vs. venous whole blood VAMS; capillary VAMS vs. venous whole blood fluid; 50 healthy volunteers.	[33]
Phosphatidylethanol	Venous whole blood	LC-MS/MS	Table S4	Comparison of venous whole blood VAMS analysis between two laboratories; 59 individual donors.	[34]
Phenylalanine, Tyrosine	Venous whole blood	Flow injection analysis-MS/MS	Table S5	Comparison of three VAMS devices (Hemaxis-DB10, Neoteryx-Mitra, and Capitainer-qDBS device) and conventional DBS.	[35]
Phenylalanine	Capillary finger blood and plasma.	LC-MS/MS		Comparison of capillary VAMS vs. plasma fluid; 24 patients.	[36]
Phenylalanine, Tyrosine, Homogentisic acid	Urine	LC-MS/MS		Comparison of urine VAMS and urine fluid.	[37]
24 Amino acids, 12 Organic acids	Venous whole blood.	LC-MS/MS	Table S6	Comparison of spiked and unspiked venous whole blood VAMS.	[38]
5 Amino acids, 4 Organic acids	Mouse whole blood	LC-MS/MS		Not performed	[39]
24 Tryptophan pathway metabolites	Mouse whole blood	LC-MS/MS	Table S7	Comparison between blank and fortified whole blood VAMS samples from healthy mice; real samples from 3 mice.	[10]

Abbreviations: enzyme-multiplied immunoassay test (EMIT); creatine oxidase method (COM).

## 4. VAMS Application Studies on Endogenous Metabolites

### 4.1. Creatinine

Given its predominant clearance by renal filtration, creatinine is the biomarker of choice to assess this organ’s function and is of crucial importance in cases of renal transplants [40]. In these patients, the monitoring of antibiotics and immunosuppressants is also of the utmost relevance. Currently, the quantification of these compounds relies on samples taken by venipuncture. However, new efforts are being made towards developing a microsampling strategy that can quantify both endogenous and exogenous analytes in a single analysis. That is why, so far, all the published methods employing VAMS technology for this purpose have been developed with the aim to simultaneously determine creatinine and administered drug levels to perform therapeutic drug monitoring of these patients while assessing their renal function.

In the work published by Andriguetti et al., both creatinine and the antibiotic vancomycin were analyzed in plasma sampled with VAMS [21]. The authors managed to develop and validate a method for the reliable quantification of both analytes; however, as endogenous compounds are within the focus of the present study, only the analytical figures of merit for creatinine are provided in Table S1. For the clinical validation of the methodology, they compared the results obtained with VAMS samples collected from plasma with those obtained through direct plasma analysis without the employment of microsampling. Both types of samples were analyzed by LC-MS/MS, and method comparison was performed by means of Passing–Bablok (PB) regression and Bland–Altman plots. The analyzed samples were collected from routine vancomycin measurements at Hospital São Vicente de Paulo (Passo Fundo, Brazil). No further details were provided on the condition of the patients. The absolute creatinine concentrations (n = 46) obtained through VAMS were, on average, 17.1% higher than those obtained by the standard sampling approach. To correct this bias, the concentration obtained through VAMS methodology was modified using the Passing–Bablok equation or a correction factor, resulting in all estimated concentrations being within ±25% of the reference values in all cases. In other studies, creatinine was determined simultaneously with tacrolimus, the most commonly used immunosuppressant to avoid transplant rejections, using VAMS in patients who had undergone renal transplantation. Mathew et al. [22] and Marshall et al. [23] presented validated methods for the quantification of these analytes. Marshall et al. conducted a comparison between the creatinine results obtained on finger-prick VAMS and standard venous samples obtained through venipuncture from 135 renal transplant patients [23]. Both were analyzed by LC-MS/MS. They found a good correlation throughout the test range for the microsampling vs. venipuncture approaches using the PB regression and a mean bias of −6.5% was found on Bland–Altman plots. The chromatographic approach was also compared with an enzymatic creatinine assay. To do so, 43 samples were analyzed using both methods without employing VAMS, using conventional sampling methods to obtain the serum concentration. The PB analysis resulted in an acceptable correlation, and the Bland–Altman bias was 0.3%.

Mathew et al. also performed a clinical validation of the developed method [22]. The study included 152 renal transplant patients. The researchers compared the results of fingerpick VAMS against samples obtained by venipuncture. Additionally, the same comparison was made using DBS analysis instead of VAMS. It was demonstrated that the Mitra devices have better predictive results than DBS analysis for creatinine analysis. The data obtained from the 152 patients was divided into two groups, a model group and an independent validation group. The creatinine data obtained with VAMS devices and LC-MS/MS analysis were also compared to reference serum values obtained by a standard creatininase–sarcosine enzymatic assay. The PB regression results indicated a significant proportional and constant bias. The Bland–Altman plot revealed a mean bias of −21.57% for creatinine in VAMS venous blood compared with the serum creatininase–sarcosine enzymatic assay. The PB equation obtained with the model group was used to correct the results obtained in the validation group. After correction, the absolute creatinine mean bias was 0.002 mg·dL^−1^, with 81.6% of the samples within a ±15% absolute deviation from the serum reference results.

Zwart et al. [24] and Wang et al. [25] also independently validated methods for the quantification of creatinine concurrently with drugs for monitoring renal transplant patients. Zwart et al. clinically validated a method for blood determination of tacrolimus and creatinine in renal transplant patients, as well as the immunosuppressant drug mycophenolic acid and iohexol, a chemical agent used for X-ray imaging whose plasma clearance has recently gained attention as it can be used to calculate the glomerular filtration rate (GFR) and thus assess renal function [24]. The method proposed by Wang et al. can also quantify the same analytes, with the exception of iohexol [25]. These methods have been validated for endogenous creatinine with satisfactory figures of merit, as represented in Table S1.

Zwart et al. compared the results from 25 patients who had undergone kidney or kidney–pancreas transplants over a year before the study [24]. All patients were receiving tacrolimus as an immunosuppressive therapy and had a creatinine clearance higher than 25 mL/min/1.73 m^2^. Reference serum creatinine concentrations were obtained through means of a creatininase–sarcosine enzymatic assay and fingerpick VAMS samples were collected in duplicate. This method showed good results for creatinine prediction via VAMS, with a mean bias of −2% (range: −22 to +24%) and 92.0% of the creatinine results being within 20% agreement with the reference ones. The authors also calculated the glomerular filtration rate (GFR) through the CKD-EPI formula, obtaining a +4% (range: −24 to +34%) mean bias for the value calculated with VAMS results against the reference serum values. For the GRF, 80.0% of the VAMS results were within 20% agreement with the reference ones.

Wang et al. compared the results obtained from capillary blood sampled through a fingerpick using VAMS devices with whole blood samples [25]. The VAMS creatinine levels were obtained using the described LC/MS-MS method, whereas the creatinine concentration in the blood obtained through venipuncture—used as the reference—was determined using the creatine oxidase method. A total of 40 samples (24 males and 16 females) from patients who had undergone kidney transplantations at different times (from 60 to 394 days prior to analysis) were compared. The patients’ ages ranged from 27.5 to 38 years. As performed in other studies, the method comparisons were defined using Passing–Bablok regression, whereas biases were measured using Bland–Altman plots. The creatinine serum concentration range was 115.50 ± 33.03 nmol/mL, whereas the VAMS results were lower, around 61.14 ± 22.93 nmol/mL. Such results were corrected using the equation obtained with PB regression, resulting in concentrations that had good correlation with the serum reference results on the PB plot, with no significant bias (−1.50%) found in the Bland–Altman analysis. A total of 92.50% of the corrected VAMS results were within the established clinical acceptance limit of ±15% absolute deviation from matched samples.

Lastly, Scuderi et al. also published a method for the quantification of serum creatinine and tacrolimus [26]. Their study focused more on the statistical comparison of the results obtained through venipuncture and VAMS in clinical samples. Both VAMS and serum samples were analyzed using the same UPLC-MS/MS setup. The study analyzed 37 patients who had undergone kidney or kidney–pancreas transplants and were taking tacrolimus, who had had a stable dose for at least the last 7 days. Pregnant or lactating patients, or those diagnosed with hepatitis B, hepatitis C, or HIV were not included in the study. The PB regression of creatinine displayed a −35% under measurement in VAMS compared with the serum results. The values were corrected with PB regression, resulting in no significant proportional or constant bias, as shown by a Bland–Altman percentage difference of 1.42% with a 95% limit of agreement of −34.1 to 36.9%. All the aforementioned results can be seen in Table S1.

Thinking from an application point of view, the sensitivity achieved by the methods seems to be satisfactory. As is presented in Table S1, the limit of quantification (LOQ) in all the methods was below the normal ranges found in adults (0.7 to 1.3 mg/dL for adult males and 0.5 to 1.1 mg/dL for adult females). The applicability of the methods was also proven by the clinical studies performed, all of which demonstrated that VAMS devices can be employed for creatinine determination in real samples, although a correction of the obtained results was necessary to compensate for the bias obtained with the microsampling approach. Andriguetti et al. observed that this trend was more pronounced at higher creatinine concentrations, which coincides with the higher extraction yield obtained for the more concentrated quality control samples in method validation [21]. Therefore, before applying this microsampling strategy in the clinical framework, one must be aware that the real values must be corrected according to the results of the clinical validation performed beforehand.

Overall, we can say that the applicability of VAMS devices for the determination of creatinine in blood has been successfully validated by the different authors. A more complete overview of the analytical validation parameters can be seen in Table S1.

### 4.2. Steroids

In the past decades, steroid analysis has gathered much attention due to its application in clinical environments, as well as its implications in sports for the detection of performance-enhancing substances. The most relevant substances used as drugs of abuse in doping are androgenic anabolic steroids (AAS). The use of doping is usually established when a substance on the WADA (World Anti-Doping Agency) Prohibited List is found [41] or when the levels and/or ratios of certain endogenous steroids surpasses a given limit [42]. In the framework of an athlete’s biological passport [43], a longitudinal study of the individual athlete is performed, establishing personal ranges within which the levels of steroid hormones can physiologically vary. In this context, microsampling techniques have emerged as a powerful tool due to their non-invasive nature and their increased stability, which is greatly advantageous for the long-term storage of the multiple samples required for anti-doping analysis.

Chang et al. developed a method for the quantification of an endogenous AAS (testosterone) and eight exogenous compounds (used for doping) in blood samples collected via VAMS by GC coupled to a triple quadrupole mass detector [27]. Although low recovery and significant matrix effects were observed for some of the exogenous analytes, the method achieved satisfactory results for testosterone, as shown in Table S2. The agreement between the testosterone levels determined via VAMS and those in serum was assessed by Deming regression and Bland–Altman analysis, with no relevant variation between the methods. A three-arm clinical study, using 21 male participants aged from 18 to 45 years old, demonstrated the capacity of the method to detect the use of exogenous testosterone gel in microdoses, despite the intrinsic sensitivity limitation of the VAMS devices.

The suggested methodology can be considered to have some limitations, as the use of GC-MS/MS requires derivatization and urine, rather than blood, is the usual matrix chosen by WADA for steroid analysis due to its high concentration of steroids and metabolites. Such aspects were considered by Protti et al. in the development of an LC/MS-MS method for the quantification of unconjugated anabolic androgenic steroids in urine [28]. The applicability of VAMS devices, which are designed to collect a fixed volume of blood, was tested gravimetrically for urine specimens and obtained satisfactory results towards the volume sampled, with a slightly higher deviation (±0.86) in comparison with pipetting a volume of 30 μL (±0.25). The data acquired by the VAMS method—which analyzed four endogenous steroids (testosterone, epitestosterone, DHT, and dehydroepiandrosterone (DHEA)) and nine exogenous compounds—was compared with those obtained in fluid urine samples using Passing–Bablok regression, resulting in comparison slope coefficients close to the unity (ranging from 0.9942 to 1.0131), regression coefficients greater than 0.9991, and negligible intercepts. Similar results were obtained for dried urine spot (DUS) analysis—an analog to DBS analysis but in a urine matrix—in the same study.

The analysis of endogenous AAS was also performed in blood samples using VAMS devices by Marshall et al. [30]. The authors developed an LC-MS/MS method for the quantification of testosterone, androstenedione, and 17-hydroxyprogesterone. The validation parameters are presented in Table S2. In this methodology, analyte specificity was tested by a panel of structure-related compounds, all of which presented no relevant interference with the test analytes. Hemoglobin (47 g/L), bilirubin (600 mg/L), and intralipid (172 mL/mL) were also tested as interfering substances to check whether their presence would cause an alteration higher than 5%; this did not happen for any of the studied compounds. The analytes were found to be stable for 14 days at room temperature and 200 days in the freezer (−20 °C) in the VAMS devices.

Comparative assays were also performed in order to assess the applicability of VAMS devices for such purposes. To do so, VAMS results were compared with serum and hematocrit-corrected plasma results from the same sample. Passing–Bablok regression showed good correlation for the plasma comparison (testosterone (R^2^ = 0.96), androstenedione (R^2^ = 0.99), and 17-hydroxyprogesterone (R^2^ = 0.99)); however, a proportional bias was present, requiring the application of correction factors. Regarding the serum comparison, minimal bias was shown for androstenedione and 17-hydroxyprogesterone but a significant bias of 4.28 nmol/L for testosterone was present.

Due to their reduced invasiveness and use of small sample volumes, VAMS also represents a convenient tool for animal studies, as demonstrated in the work of Heussner et al. in the study of steroids in rats [29]. In this work, VAMS devices were used to analyze 11-dehydrocorticosterone, cortisol, testosterone, and progesterone in rodent blood samples; the results were then compared with EDTA plasma levels. The study also compared the results from different microsampling devices, i.e., Whatman^®^ 903 Protein Saver Cards, Noviplex^TM^ Plasma Prep Cards, and the Mitra^TM^ microsampling device (VAMS). It should be noted that a previously published method for steroid analysis in placenta was used to perform the analysis, but the validation parameters to assess the applicability of this methodology for this study were not present in the article [44].

One of the factors assessed in the study was the hematocrit effect. Thus, different approaches were adopted: first, pooled rat blood was spiked with different cortisol concentrations. In addition, pooled human blood (n = 5) and Wistar rat blood (n = 8, 18 weeks with mixed gender) glucocorticoid concentrations were analyzed and the hematocrit effect was determined by comparison with EDTA plasma results. A relevant hematocrit effect was observed in all the microsampling devices. To overcome this limitation, hematocrit correction factors were established and validated in juvenile (21 days, n = 11) and adult (18 weeks, n = 6) Wistar rats. Using the correction factors obtained on the hematocrit effect test, the recovery of the internal standards was tested for corticosterone-d8 and cortisol-d4 by comparing the glucocorticoid levels obtained with the microsampling devices with those obtained with EDTA plasma from the same sample. Every tested microsampling technique obtained comparable results for all the analytes, except for Noviplex^TM^ Plasma Prep Cards, which displayed a considerable analytical loss of corticosterone-d8. The VAMS devices obtained, among the microsampling approaches tested, the lowest limit of quantification. The coefficient of variation (CV%) results in intra-day precision analysis using these devices varied from 18.0% to 32.0%. The aforementioned results highlight some factors that need to be taken into consideration when applying microsampling techniques to animal studies. First, the hematocrit effect was relevant in all the different microsampling approaches and must be assessed for each analyte during the method validation. Secondly, the type of device itself might affect the sampling amount of the analytes differently, as occurred with corticosterone-d8 using the NoviplexTM device.

The analysis of steroids was in all cases performed on a triple quadrupole MS system; in all studies, spiking of the samples prior to sampling with the VAMS devices was performed. Electron impact (EI)–GC was used [27], or LC with either APCI [29] or ESI source operating in positive ion mode [28,30]. Overall, in most of the studies acceptable analytical figures of merit were obtained for the VAMS methodology, except for the study by Heusnner et al., which did not present full validation results [29]. Despite the evident drawback of lower sensitivity when using small sample volumes, the methods could reach a limit of quantification (LOQ) low enough to sufficiently cover the concentration of these endogenous compounds in real samples. Moreover, it should be noted that some methodologies involve preconcentration of the extract prior to analysis. However, the methodology of Chang et al. is not sensitive enough to analyze the normal levels of testosterone in female subjects [27]. The stability results varied depending on the methodology, with Chang et al. and Protti et al. displaying a decrease in the concentration of the endogenous analytes below 20% after 2 months [27] and a year [28] of storage at room temperature, respectively, whereas Marshall et al. only showed a stability of two weeks under these conditions [30].

### 4.3. Vitamins

Vitamins are small molecules that play a major role in several biological functions, participating in processes such as growth, the immune response, and others. Most of those essential compounds cannot be synthesized by the human body, so their levels are highly dependent on nutrition. Their determination in the blood is crucial to assessing the health status of a patient, as vitamin deficiency is associated with several health issues. Blood microsampling techniques could greatly aid in such determinations and be applied in the frequent assessment of vitamin deficiency, diet, or the effectiveness of supplementation, thus improving health monitoring in the population.

Vitamin D (25-hydroxyvitamin) deficiency is related to reduced bone mineral density [45] and plays a role in the immune, cardiovascular, and inflammatory systems. The vitamin D receptor (VDR) is widely expressed throughout the body, being present even in tissues not involved in calcium and phosphate transport. As well as that, a considerable part of the human genome is under its direct control. Great interindividual variation between the vitamin D levels in the blood is often observed, as diet and sun exposure are the two main factors related to its intake [46].

Tuma et al. developed a method for the quantification of hydroxyvitamin D2 (25(OH)D2) and D3 (25(OH)D3) in blood by employing VAMS devices [31]. In their study, a purification procedure was employed to obtain blank blood samples for method validation. In it, the whole blood serum was separated from the red blood cells, which were then washed with PBS three times to remove any trace of 25-hydroxyvitamin. Reconstitution to a HCT level of 40% was achieved by mixing the cleaned red blood cells with human serum albumin. Lyophilized serum controls were reconstituted to a HCT of 40% to achieve the QC standards, whose final concentrations were 15.1 and 49.1 ng/mL for 25(OH)D2 and 14.5 and 53.6 ng/mL for 25(OH)D3.

The VAMS extraction procedure was performed using methanol containing the deuterated internal standards of d6-25(OH)D3 in an ultrasonic bath for 30 min. The extract was evaporated and the dry residues were reconstituted in 60 μL of a methanol:water mixture (80:20 *v*/*v*) before being injected into a UHPLC-HRMS system.

Satisfactory validation results were obtained for both the 25(OH)D2 and 25(OH)D3 analytes, as can be seen in Table S3. The matrix effect resulted in a recovery of 81% for 25(OH)D2 and 70% for 25(OH)D3, showing a relevant ion suppression effect that could be overcome via correction with IS. None of the tested parameters (time in the ultrasonic bath or temperature of the vacuum centrifuge) had any influence on the overall yield of the analytes, as shown by the robustness assays, whereas the HCT effect was not assessed in this study.

Regarding stability, no degradation was observed when the VAMS devices were stored at −18 °C for 28 days. At room temperature, a decrease of less than 8% for 25(OH)D2 and less than 3% for 25(OH)D3 was observed in the same timeframe, which falls within the method’s variation. In contrast, fluid samples at room temperature displayed a relevant degradation after only 3 days, being reduced to 38 and 50% of the respective original concentrations of 25(OH)D2 and 25(OH)D3 by the end of the 28 days. This demonstrates that the analyte’s stability was greatly improved by the VAMS devices.

The applicability of the method in clinical practice was explored by analyzing blood samples from 20 healthy adults at the end of the summer in Germany. 25(OH)D3 was found in all samples, with concentrations that ranged from 6 ng/mL to 60 ng/mL, whereas 25(OH)D2 was detectable only in one sample. The results were within the expected range, since vitamin D shows high interindividual variability, with the levels ranging from deficiency (<10 ng/mL) to optimal (>20 ng/mL) concentrations [47]. It should be highlighted that no comparison with conventional sampling methods was performed. Nevertheless, the presented data are in agreement with a previous study reporting the comparison of vitamin D measurements in DBSs and serum [48].

Another micronutrient whose deficit is related to several health conditions is thiamine, also known as vitamin B1. Thiamine diphosphate (TDP), the most relevant thiamine derivative, acts as a co-factor for several enzymes involved in energy metabolism, and its deficiency causes the disease commonly known as beriberi. Verstraete et al. developed a method for the quantification of TDP in blood sampled using VAMS devices [32,33]. The authors compared the thiamine results in venous blood samples with those acquired through capillary and venous blood sampled using VAMS. For sample preparation, the VAMS polymeric tips were extracted with 150 μL 12% TCA containing D3-TDP (5 ng/mL) as IS and the analysis was performed on a UPLC system coupled with a triple quadrupole MS and an ESI ion source operating in positive ion mode. For the preparation of whole blood samples, the red cells were lysed at −80 °C to release thiamine. An aliquot of 50 μL of venous blood was extracted using 400 μL of 12% TCA, also containing the IS, for 10 min at 1000 rpm at 25 °C.

The validation results obtained were within the acceptable limits, as can be seen in Table S3. Reconstituted blood was used as a QC standard and analyzed together with native blood samples. Precision measures—tested in both whole blood and the dried matrix—were below 13% and 8% for the QC and native samples, respectively, as calculated by the ANOVA test. The accuracy study showed bias up to 6.5% for both matrices. Likewise, Tuma et al. reported that for vitamin D [31], the matrix effect test showed relevant ion suppression throughout a broad range of HCT, which was corrected by the application of IS. No impact of HCT was found, with all samples varying within a 15% limit when normalized to normal HCT levels. VAMS samples could be stored for up to 7 days at −60 °C. At room temperature, the samples proved to be stable for up to one month and were shown to be stable under light, with no need for storage in dark places. Whole blood samples were stable for 12 h at 37 °C and for 24 h at room temperature, indicating the superiority of VAMS regarding storage.

A test for clinical applicability was performed in 50 healthy volunteers. For each volunteer, three blood samples were collected: venous whole blood samples, venous blood VAMS samples, and capillary blood VAMS samples. Firstly, the correlation between the venous whole blood and the venous blood sampled using VAMS was assessed through a Bland–Altman plot and Deming regression analysis. A good correlation between methods was found, with a statistically non-significant mean bias of −0.7% in the VAMS samples; no systematic or proportional differences were observed. Additionally, the same comparison procedure was performed between venous blood and capillary blood sampled using VAMS. The Bland–Altman plots showed a non-significant mean bias of −1.0%, whereas Deming regression showed no systematic or proportional differences.

### 4.4. Phosphatidylethanols

Phosphatidylethanols (PEths) are a group of phospholipids that have gained more attention in the past few years due to their applicability in the monitoring of alcohol consumption. They are formed by the enzyme phospholipase D when ethanol is present in the bloodstream [34]. This reaction physiologically uses H_2_O to hydrolyze its substrate, phosphatidylcholine, into phosphatidic acid (PA) and choline. However, when primary alcohols such as ethanol are present, H_2_O is replaced and a transphosphatidylation reaction occurs, giving PEth instead of PA [49]. More than 40 PEth analogues have been found in the blood of heavy alcohol consumers; however, PEth 16:0/18:1, PEth 16:0/18:2, and PEth 18:1/18:1 are the predominant ones, with 16:0/18:1 being the most abundant.

Given the relevance of this metabolite in toxicological analysis, Uytfanghe et al. developed a methodology to incorporate VAMS into the determination of PEth 16:0/18:1 [34]. The LLE extraction efficiency was evaluated at two concentrations and at three HCT levels, giving satisfactory values. A deviation higher than 15% was observed at lower HCT levels when compared with those obtained at normal HCT levels. However, the authors highlight that, as the method is expected to be applied to samples with normal HCT values, such a bias will not have an impact on the analysis. The complete list of the validation parameters studied can be found in Table S4.

To evaluate the method further, an inter-laboratory comparison was performed by two different labs [34,49]. First, pre-validation experiments were performed to assess the initial differences between the methods. To do so, internal quality control (IQC) samples, prepared from the spiking of blank blood samples, were used. Additionally, external quality assurance (EQA) samples were prepared by diluting external blood samples with known PEth concentrations with blank blood. A stock solution at a concentration of 2000 ng/mL was also analyzed by both labs. After pre-validation was performed, a set of 59 blood samples from different volunteers were analyzed in both laboratories and the results were analyzed using Passing–Bablok regression and Bland–Altman plots.

A comparison of the validation parameters from the two labs shown in Table S4, suggesting similar levels for the precision and accuracy of the method at the different sites. Based on the analysis of the samples in the pre-validation steps, it was observed that although both labs made use of the same extraction procedure, the use of distinct instrumental settings gave rise to a difference of 30% in their data. Initially, the reason for this bias between the EQA samples was thought to be due to the equilibrating time of the spiked blood samples prior to the application on the VAMS devices. As the analyte is found at the surface of red blood cells, an equilibrating time would be necessary for the incorporation of PEth into the sample. However, this practice did not improve the reproducibility of the assays.

This error could be explained by the fact that the ratio of PEth 16:0/18:1 to 18:1/16:0 isomers in the reference material (the EQA samples) was different from the naturally occurring ratio and by the fact that both analytes have a different fragmentation efficiency for their side chains [50]. As both analytes were not chromatographically separated and were thus being quantified at the same time, those factors could be the source of variation in this transition.

After harmonizing the protocol, the results between the two laboratories varied only by an average difference of 1%, with an SD of 10%. Based on the analysis of 59 blood samples from different volunteers, a satisfactory correlation between the data was found, as the slope and intercept were not significantly different from 1 and 0, respectively. No bias (−0.4%) and a rather narrow confidence interval in the Passing–Bablok curve were found. The sensitivity found by both labs seems to be compatible with the blood concentrations of the analyte, since the results were above the LOD (1.7 ng/mL) for 54 out of the 59 samples in one lab and 51 were above the LOQ (10 ng/mL) in the other. The matrix effect was evaluated and found to be relevant when no IS correction was performed, with 79% and 114% recovery, respectively. After IS correction was applied, the respective average recovery for the matrix effect was 98% and 109%. The percentage RSD values were also improved with the use of IS, as they were reduced to 8%. The stability study demonstrated excellent results, with the samples being able to support storage at room temperature for 400 days, with the vast majority of samples (87%) presenting a decrease smaller than 15.0%.

The comparison between the two labs highlights the need for robust and detailed analysis protocols to ensure the reproducibility of the data, as many factors may influence the final result. Overall, the method has been successfully validated in a cross-laboratory investigation. Both instrumental setups presented satisfactory figures of merit, and the proposed sample treatment methodology was proven suitable for the analysis of PEth using VAMS devices.

### 4.5. Aminoacids and Other Metabolic Biomarkers

As there is a high need for frequent monitoring in patients with metabolic disorders, an interesting application of VAMS is in the assessment of these conditions by measuring one or more key intermediates of important metabolic pathways.

An example is the assessment of phenylketonuria (PKU) through the measurement of phenylalanine (Phe) and other related metabolites in the blood. PKU is caused by a mutation that affects the conversion of Phe into tyrosine (Tyr), resulting in the accumulation of Phe, which can lead to several types of brain and nervous system damage [51]. PKU screening is exceptionally relevant in newborns to immediately detect the condition before any symptoms appear, making the use of microsampling approaches even more pertinent.

Carling et al. developed a method for the quantification of Phe and Tyr for PKU screening in blood samples collected with different microsampling techniques [35]. Tyr’s analysis is relevant in the context of PKU monitoring to assess whether the patients are becoming Tyr deficient due to their synthetic diet and lower conversion between the two amino acids. In this study, different DBS approaches and VAMS were compared with two other microsampling techniques: Hemaxis and Capitainer-qDBS devices. The latter two techniques are variations of conventional DBS analysis, where an exact volume of blood is sampled and applied to a DBS card, thus overcoming the drawbacks of non-quantitative sampling and the HCT effect.

For the preparation of the DBS samples, 20 mL of blood was collected from a healthy adult volunteer into lithium heparin tubes, 20 uL of which was applied to conventional DBS cards (PerkinElmer-226 filter paper with a nominal thickness of 0.54 mm) or to PerkinElmer-226 Bioanalysis RUO cards (the same filter paper with four pre-perforated 8 mm discs to which the blood was applied). A 3.2 mm sub-punch from the DBS was collected and placed into a 96-well plate. The extraction solvent, methanol containing stable-isotope-labeled (SIL) Phe and Tyr internal standards, was added (the same for all but different volumes) and the plate was agitated in a shaker for 35 min. Liquid whole blood samples (10 μL) were also analyzed; however, methanol was replaced by acetonitrile to ensure adequate protein precipitation. The authors reported that replacing methanol with acetonitrile did not affect the mean measured concentration or variance for both analytes (n = 10; *p* > 0.01).

The samples were analyzed using UHPLC-MS with ESI in positive ion mode. The first parameter studied was the effect of different blood volumes sampled on the Phe and Tyr analyses. The VAMS polymeric tips were not affected by the volume of blood the tip was put in contact with, as the tip was saturated after sampling the exact volume it was designed for. Similarly, the application of different volumes of blood on the Hemaxis and Capitainer-qDBS devices did not affect the concentrations of the analytes. However, for conventional DBS sampling, applying a volume of 20 or 50 μL greatly influenced the analyte concentrations, with biases for Phe and Tyr of 16.6 and 13.6%, respectively (*p* < 0.01). This highlights one of the many advantages of employing volumetric microsampling devices instead of conventional DBS analysis.

The HCT effect was determined by measuring samples with three HCT levels (29.2, 44.4, and 63.3%). It was found that the Phe and Tyr concentrations varied significantly at different HCT levels on the VAMS devices, as can be seen in Table S5. This effect was found to be more relevant at higher HCT concentrations. The same effect occurs in conventional DBS analysis, where a significant variation in the concentration of both analytes was demonstrated when compared with liquid blood samples. The other microsampling approaches, including PerkinElmer-226 Bioanalysis RUO cards, gave rise to an acceptable bias that was lower than 10%.

Accuracy and precision were studied in 10 replicates at three different concentrations. Bias was determined by comparing the obtained results with those obtained using liquid whole blood analysis. The results are presented in Table S5. Conventional DBS analysis presented an overall bias higher than the acceptance limits (±10.4 for Phe and ±15.5% for Tyr), whereas the other two microsampling approaches (Hemaxis and Capitainer-qDBS) presented deviations from the acceptance limits. Both conventional DBS and VAMS analyses were found to be more precise than liquid blood analysis, as determined by an f-test (*p* < 0.01), whereas the other microsampling approaches did not show any difference from liquid blood in terms of precision.

Regarding stability, both analytes were stable for 15 days at both room temperature and 4 °C after sampling with VAMS. A recognized limitation of this study is that no clinical validation of the method was performed.

Another study that applied VAMS in the framework of Phe analysis for detecting PKU was performed by Gao et al. [36]. The authors performed a correlative study comparing Phe measurements in whole blood sampled with VAMS against liquid plasma. To overcome the limitation of validating a method quantifying an endogenous analyte in a biological matrix, surrogate isotopes of Phe (2,3-13C2,15N-phenylalanine) were used. Additionally, a standard addition method using Phe itself was also performed, correcting the concentrations of the analyte for those obtained by the analysis of non-spiked plasma.

Extraction was performed using an aqueous IS solution (^13^C_9_,^15^N-phenylalanine) at a concentration of 150 μmol/L containing 0.1% formic acid. Subsequently, acetonitrile:methanol (50:50, *v*/*v*) was used to perform protein precipitation. The supernatant was diluted with 0.1% TFA in water and analyzed using HPLC-MS/MS.

The validation data are presented in Table S5. Selectivity was assessed only for the isotope surrogate in six different samples of whole blood sampled with VAMS, as well as with lipemic blood (turbid blood samples due to the presence of lipoprotein particles) and whole blood at three different HCT levels. No interference with the isotope surrogate was detected. The recovery (measured in six replicates at three different concentration levels) ranged from 88 to 108.4%. The matrix effect was determined for the surrogate isotope with a bias between −13.5% and 4.9% and a % CV between 1.2% and 12.6%. The authors also assessed the matrix effect using Phe external standards, and no significant matrix effect for accuracy or precision was reported.

The HCT effect was assessed in triplicate at three different HCT levels and at two different Phe concentrations and had a bias ranging from −10.7% to 3.1% and a % CV from 2.5% to 9.3%. The lipemic effect (alteration of the analytical response due to the presence of lipoprotein particles) was assessed by spiking the Phe isotope into lipemic whole blood samples at different concentrations. The mean bias found was between 0.4% and 3.6%, with the respective % CV between 1.9% and 7.9%.

Phe was shown to be stable in VAMS devices for 232 days at room temperature and for at least for 15 days at 45 °C, whereas the whole blood analyte was stable for only one hour at room temperature and 24 h in plasma at room temperature.

The results obtained with VAMS analysis were also compared with the liquid plasma ones. The intra- and inter-day precision and accuracy of both methodologies were found to be equivalent, with deviations within the acceptance criteria of 15%.

The developed method was applied to samples from 24 PKU patients who were divided into six groups and treated with three different therapies for lowering Phe levels (PTC923 20 mg/kg/day, PTC923 60 mg/kg/day, or sapropterin dihydrochloride 20 mg/kg/day). The treatments took place over a 7-day period, with 7-day washout periods in between. Whole blood and VAMS samples were collected on different days before and during treatment, as well as in the wash-out phases.

The Pearson correlation coefficient of the 296 appropriately collected matching samples was 0.9813, showing a significant and strong positive correlation. Passing–Bablok analysis gave an intercept close to 0 and a slope close to 1, indicating negligible systematic and proportional differences. A Bland–Altman analysis conducted based on proportional differences showed that the data were equally distributed around the mean value. For ISR analysis, the two methods were considered equivalent when a minimum of 67% of the matching samples presented a deviation up to 20%. Phe analysis in VAMS and in liquid plasma, at three concentration ranges, generated at least 84.0% of results that deviated less than this established threshold. Therefore, ISR analysis indicated no systematic difference between the two compared methods.

It seems that VAMS, as previously discussed in steroids applications, can be important for use in urine analysis as well. Taylor et al. performed a comprehensive study on the use of VAMS for the determination of Phe, Tyr, and homogentisic acid (HGA) in urine for the diagnosis and monitoring of another metabolic disorder, alkaptonuria [37].

Alkaptonuria is a congenital metabolic disorder caused by a dysfunction in Tyr metabolism. Tyrosine is converted into 4-hydroxyphenylpyruvic acid, which is then metabolized into HGA. Due to an error in the enzyme homogentisate-1,2-dioxygenase, this last metabolite accumulates in the body, where it undergoes oxidation and polymerization into melanin-like pigments that deposit inside and outside the cells. This leads to weakening, stiffness, and malfunction of the tissues, with symptoms starting to appear after the third decade of life [37].

VAMS devices offer an advantage in the framework of alkaptonuria due to logistical, financial, and patient-related concerns. Hence, a method previously validated for the quantification of Tyr and HGA in liquid urine samples was applied to VAMS by also adding Phe as an analyte [37]. The adapted method was fully revalidated; however, the validation data were not provided.

The extracts were analyzed using a UHPLC-MS system with ESI operating in positive ion mode for Tyr and in negative mode for HGA. The ionization mode for Phe quantification was not presented, as the validation data were not provided. Deuterated IS of Tyr and Phe were employed, whereas the IS for HGA consisted of a C-13 labeled HGA (13C6-HGA). The QC samples were prepared by adding stock solutions into an acidified pooled urine sample. To ensure low Phe and Tyr concentrations, non-spiked urine samples were analyzed beforehand, although no prior quantification of HGA was mentioned.

As VAMS devices were originally developed to sample blood, the authors studied the accuracy of the sampled urine volume gravimetrically using the specific density of the sampled matrix. Different sampling protocols using different contact times were tested and compared with pipetting. The mean deviation of all protocols was smaller than 5.0%, with no statistically relevant oversampling when increasing the contact time of the tip with the sample. Different extraction protocols were also tested using an acidified aqueous solution of IS as the extraction solvent. The final extraction method was set as 15 min of orbital shaker agitation, with a mean recovery of all the analytes of around 101.2% (±1.9).

The stability of VAMS was assessed in acidified and non-acidified urine samples under different storage conditions (at 4, 20, and 37 °C) for a period of 28 days. Tyr and HGA were stable only when stored at 4 °C and rapidly degraded when stored at 37 °C. Tyr’s deterioration at 20 °C was apparent only on the 14th day. A visible darkening of the VAMS devices containing alkaptonuria patients’ urine was noted, evidencing the oxidation and polymerization of HGA into melanin-like pigments even in dried matrices. Phe was stable in all storage methods for the full test period of 28 days. Acidification was shown to be unnecessary, as it only caused a slight improvement in stability for Phe, which was nevertheless considered stable even when no acidification was employed. The protection of the samples from light did improve HGA stability; however, this improvement was not enough to consider the analyte stable in any method that did not involve low temperatures.

The method was applied to the analysis of 21 urine samples from untreated patients with alkaptonuria. The results from VAMS were compared with those from urine using Passing–Bablok analysis and Bland–Altman plots. The initial results pointed to a 15% negative bias for all analytes sampled with VAMS in comparison with urine. The bias was due to comparison with the liquid standards used for quantification. In fact, when dried standards were used, the bias disappeared. HGA concentrations were all above the upper limit of quantification, so the samples were diluted to perform the comparative analysis.

Besides the targeted applications of VAMS for specific amino acid biomarkers, there have been two studies that clearly show the potential of these devices for multi-targeted assays. This includes the effort of Kok et al., who developed a methodology for the profiling of 24 amino acids and 12 organic acid metabolites in blood using a single microsample from VAMS [38].

In this work, due to the chemical differences between the two classes of analytes, two different chromatographic setups coupled to a triple quadrupole were employed. Hydrophilic interaction chromatography (HILIC) to quantify amino acids and reversed-phase UHPLC/MS-MS for organic acids. By combining the two methods, 36 metabolites could be determined in a small sample. This multiplexing of the analytical methods is very desirable in clinical laboratories, as a single analysis can cover many biomarkers, making the overall procedure much simpler and cheaper [38].

Extraction was performed using acetonitrile/water (60:40 *v*/*v*), as this was found to maximize the recovery of the analytes from the VAMS tips. The extraction process consisted of putting the polymeric tip in contact with the extraction solvent for 5 min, followed by vortex agitation for another 5 min. For the analysis of the organic acids, the extract was evaporated and reconstituted with water before injection into the UHPLC system. Such a step was not necessary for the analysis of the amino acids, which were directly injected into the HILIC system after vortex agitation.

The recovery was assessed by comparing the pre- and post-extraction spikes of the samples. Most of the amino acids were obtained with a recovery higher than 85% in all four concentrations (10, 25, 50, and 100%). The basic amino acids arginine and lysine showed the lowest average recoveries (46.5 and 68.0%, respectively). In attempts to improve this, it was found that wetting the tips with water before the sampling increased the signals of such analytes; however, this process resulted in the reduction of other analyte recoveries, as well as the reduction of the overall reproducibility of the assay, and therefore was not employed.

Regarding the organic acids, an overall lower recovery was found. The metabolites 2-oxo-glutaric acid and glyoxylic acid presented especially low recoveries (5.5 and 23.4%, respectively), although the results were reproducible across all the concentration ranges, with a CV lower than 5%. Similar to the results for arginine and lysine, pre-wetting the tips caused a rise in the signal of glyoxylic acid with a concomitant decrease in the signal of the other analytes.

The stability was assessed in short-term (26 h) and medium-term (15 days) storage at room temperature after sampling with the VAMS devices and based on the concentrations of the analytes. No difference in signal was found after 26 h in the dried matrix, whereas an average signal decrease of 84% was noticed when fluid blood was exposed to the same conditions. As well as this, the peak areas were not affected by three freeze–thaw cycles. The long-term stability was within acceptable limits for most of the amino acids, with only methionine and tyrosine presenting a decrease in concentration of more than 15% (19.2 and 15.4%, respectively). For the organic acids, however, relevant decreases in concentration were observed, with malic acid, glutathione, and uric acid displaying a relevant area decrease (−21.8, −31.8, and −22.9%, respectively) and glyoxylic acid, pyruvic acid, 3-hydroxypropionic acid, and succinic acid showing a relevant increase in the area (+36.8, +26.0, +37.9, and 46.3%). For these compounds, malic acid, glutathione, succinic acid, and uric acid were stable for only four days, whereas the other analytes were stable for up to seven days. Detailed validation and stability data for the method can be found in Table S6.

Another interesting multitargeted application was that of Protti et al., who developed a method covering the analysis of 24 metabolites from the tryptophan pathway in murine blood samples by applying VAMS [10]. Tryptophan (Trp) pathway metabolites, which play crucial roles in many physiological functions, are important biomarkers for several pathologies mostly related to the central nervous system. Its metabolites are related to several conditions, including neurodegenerative, autoimmune, and psychiatric disorders, cancer, and infection. Furthermore, Trp is used as a substrate for the synthesis of many neurotransmitters. Therefore, the analysis of Trp and its metabolites is very relevant for investigations in many areas.

The analysis made use of a HPLC on a pentafluorophenyl (PFP) column coupled to a triple quadrupole mass analyzer and an ESI source, operating in both positive and negative ion modes, depending on the analyte. Four IS (TRP-D5, kynurenic acid-D5 (KYNA-D5), serotonin-d4 (5-HT-D4), and indole-D7 (IND-D7)) were used to correct the responses of the analytes.

For the microsampling treatment, an ACN/H_2_O (65:35) *v*/*v* mixture was used for extraction. The authors reported that ACN allowed for a better extraction than methanol and that the addition of a small amount of water to the extraction solvent also led to an increase in response. However, higher percentages for water in the extraction mix resulted in pink-colored extracts with a higher matrix effect and lower analyte recoveries. After adding the extraction solvent, the samples were submitted to ultrasound-assisted extraction (UAE) at 40 kHz for 10 min and vortex-assisted extraction (VAE) for 10 min.

The validation results for each of the 24 analytes are presented in Table S7. For validation proposes, a standard addition method was performed, using neat and fortified VAMS blood samples obtained from healthy mice. To prepare the spiked samples, 10 μL of methanolic solutions of the standards and IS were added to the polymeric tip after the collection of blood and drying for one hour in ambient conditions.

Regarding accuracy, the samples were analyzed in triplicate at three different concentrations and provided high absolute recoveries (>85%) for all the analytes. The accuracy was also tested by standard addition on real samples, with an absolute recovery comparable with that obtained during method validation, ranging from 90 to 107%. The precision, assessed for three different concentrations using six replicates on two different days, showed RSD values of less than 9.6% for all the analytes. The matrix effect was within the range 87–96% for all the analytes, whereas linearity, assessed at seven concentrations in spiked blood samples, presented an r^2^ that was always higher than 0.9987. The method’s LOQ ranged from 0.1 to 25 ng/mL. When the method was applied to real samples from two male and one female rodent, it was able to detect all analytes in every sample. The concentrations ranged from 0.3 ng/mL (for 3-indole ethanol in the three rodents) to around 7620 ng/mL (Trp levels in a male rodent). The authors did not comment on whether such concentrations were within the expected range, as no comparison with conventional sampling methods was performed.

Finally, a very recent manuscript by Kapadnis et al. [39] reports the development of an LC-MS/MS method for the determination of five amino acids, isoleucine, leucine, phenylalanine, tyrosine, and valine and four organic acids, 4-methyl 2-oxo valerate, 3-methyl-2-oxovaleric acid, methylmalonic acid, and succinic acid using VAMS. The authors provide an extensive validation procedure using the surrogate matrix approach. SIL analytes were used as an IS, whereas calibration standards and batch QC samples were prepared by spiking analytes into a surrogate matrix containing 0.05% BSA in PBS buffer. The dried VAMS tips were placed into a deep-well microtiter plate containing 200 μL of the extraction solvent ACN:MeOH:H2O (45:45:10%, *v*/*v*/*v*). The efficiency of the whole extraction process was determined through a recovery that ranged between 99.4% and 84.5% at LQC and HQC, respectively. The precision, accuracy, and stability of the nine metabolites were tested in various reference materials and under different conditions. The detailed validation data can be found in Table S6**.** Regarding stability in whole blood on VAMS devices after 7 days of storage at −80 °C and at ambient conditions, the mean determined concentrations of the stability of the samples were within ±15.0% of their initial concentrations at day 0, and it was found that they were stable for up to three freeze–thaw cycles (within ±15.0%). The main aim of this work was the study of analytical parameters in pooled mouse whole blood, and no application to areal study or individual samples is provided.

## 5. Discussion

VAMS represents a suitable alternative to traditional bioanalytical sampling specimens such as plasma, serum, or urine and constitutes a reliable tool for the analysis of different endogenous metabolites.

A comparison between VAMS, DBS analysis (which is widely applied), and conventional sampling methods, i.e., whole blood or plasma obtained through phlebotomy, can be seen in Table 2. From the authors’ perspective, VAMS presents itself as the more advantageous technique from the analytical point of view. When compared with conventional sampling, VAMS provides simpler collection procedures, the possibility for storage at room temperature (in most cases), and better applicability in studies using phlebotomy-adverse patients. When compared with other dried matrix techniques such as DBS analysis, VAMS appears to solve the issue of the HCT effect, as has been demonstrated by most of the works present in this review.

On the other hand, there is an inherent increase in costs when employing such devices instead of conventional blood collection for small studies. However, as highlighted by the manufacturer [52], in large, multicenter clinical trials, the decrease in costs for the transport and storage of the devices may lead to a significant overall decrease in expenses. Additionally, it should not be overlooked that highly sensitive and state-of-the-art mass spectrometry instrumentation is required in most cases to allow the analysis of such a small volume of sample, which is not available in the majority of routine laboratories.

The data reported for several analytes are inconsistent across microsampling technologies and even for the same technology (such as VAMS). The standardization and optimization of procedures, validation of bridging studies, and performance evaluation are imperative for microsampling technologies to be successfully implemented in routine clinical care [53].

Herein, a collection of protocols destined for the analysis of endogenous biomolecules by VAMS is presented. The majority of methods reported describe full analytical validation studies, often complemented by a clinical validation study. Notably, the studies share some similarities regarding the analysis workflow and validation results. Regarding analyte extraction, the most prevalent solvents employed were methanol, acetonitrile, or water [10,21,22,23,24,25,26,28,29,31,35,36,37]. This is probably due to the fact that most of the analytes have good solubility in these solvents and are only related to the microsampling approach to a lesser extent. Conversely, Verstraete et al. employed 12% TCA in their work on vitamins [33] and, in steroid analysis, Marshall et al. [30] and Chang et al. [27] made use of MTBE as an extraction solvent. Furthermore, vortex- and/or ultrasound-assisted extraction seem to be the most widespread methods used in sample preparation. The instrumental setup was largely consistent across the studies, consisting of a chromatographic separation device (UHPLC or HPLC) coupled to a mass spectrometer with an ESI source and a triple quadrupole mass analyzer. Kok et al. [38] and Chang et al. [27] were the exceptions, employing HILIC and GC as the separation methods, respectively.

As aforementioned, the validation parameters can be found in Tables S1–S7. The linearity determination coefficient (R^2^) of the proposed methods exceeded 0.99 in all cases. Precision and accuracy varied depending on the analyte but were found to be satisfactory overall. The matrix effect was assessed in most of the studies, generally falling within the 15% variation range when IS correction was applied. Chang et al. found a matrix effect for testosterone just outside this range (116%) [27]. However, for the other exogenous steroids analyzed in the method, an even higher effect was observed. The authors proposed that such signal enhancement was due to instrumental alterations happening in the GC. Tuma et al. [31], Verstraete et al. [33], and Uytfanghe et al. [34] found relevant matrix effects only when no correction with IS was employed, highlighting the importance of using the IS-compensated response when evaluating the results.

Matrix effects in VAMS can arise from components of the matrix (blood or urine) or from the polymeric tip used for sampling the biological fluid. The choice of the extraction solvent and method can greatly influence whether this effect will be present or not. Further purification steps can be employed to overcome this problem, such as protein precipitation and purification with phospholipid and/or protein removal plates [2]. Furthermore, as VAMS allows for sample collection directly from a fingerpick, no use of anticoagulants, such as EDTA, is involved; the use of anticoagulants could be a confounding factor in the signal enhancement or suppression that often occurs in fluid blood analysis [6].

One of the main advantages of employing VAMS instead of other microsampling techniques is the possibility of mitigating the HCT effect, which is extremely relevant in techniques such as conventional DBS analysis. Theoretically, VAMS devices should be able to overcome this limitation due to their capability to sample an accurate, homogenous amount of biological fluid [2]. In most cases, the HCT effect was mitigated when employing this microsampling technology, with variations smaller than 20% on different HCT levels. Nevertheless, Chang et al. [27] reported a significant HCT effect when evaluating glucocorticoids levels in rat blood. Andriguetti et al. [21], Tuma et al. [31], and Chang et al. [27] did not evaluate the HCT effect in their studies, nor did Protti et al. [10] or Kok et al. [38] in their multitargeted analysis. Taylor et al. [37] and Protti et al. [28] performed analysis on urine samples.

With regard to method validation using VAMS for endogenous analytes, a valid concern is how to produce the reference material. There are three options to perform the standard addition: spike the sample prior to the sampling on VAMS, add the standard into the polymeric tip, or add the standard to the extraction solvent. The earlier into the preparation procedure the spiking process is performed, the more comprehensive the study and the representative results will be, as this assesses all the possible variation sources from the beginning and better simulates the application under real conditions. Therefore, fortifying whole blood before sampling with VAMS seems to be the most accurate way to perform method validation. This was the case for Andriguetti et al. [21], who sampled spiked plasma with VAMS devices. Additionally, Marshall et al. [26] and Mathew et al. [25] added their internal standards to whole blood samples obtained by venipuncture before sampling with VAMS and Protti et al. [28] did the same on urine samples for steroid analysis. However, this brings up the necessity of collecting biological material and the need for trained personnel to perform this procedure. Alternatively, adding the standards to the extraction solvent seems to be the most used alternative. This was the strategy performed by Wang et al. [25], Zwart et al. [24], and Scuderi et al. [26] in their creatinine analyses and by Kok et al. [38] on the quantification of amino acids and organic acids. Lastly, the addition of the internal standards to the polymeric tip before sampling seems to be a risky approach, as mentioned by Protti et al. [2], for it could lead to modification of the tip and affect the sampling of the biological matrix itself. None of the mentioned works made use of such a spiking approach.

An important feature that is questionable for this type of sample and thus needs to be studied in such applications is the stability of the analytes. Theoretically, the dried matrix would decrease the analyte’s deterioration, allowing storage for longer times and in milder circumstances, i.e., ambient conditions. Interestingly, most of the studies displayed an improvement in stability when compared with fluid samples, although some analytes still display fast deterioration in ambient conditions. The increase in stability is highly dependent on the nature of the analyte. For instance, Tuma et al. [31] showed that VAMS increases the stability of vitamins by about 10-fold at room temperature, whereas Taylor et al. did not find any noticeable changes in the stability of Tyr on VAMS devices.

Finally, it has been shown that VAMS can be effective in sampling biological fluids other than blood. Protti et al. [28] determined gravimetrically that the urine volumes sampled with VAMS have similar precision to the blood volumes sampled and even higher precision than pipetting. A difficulty that arises from employing alternative matrices is assessing when the complete saturation of the tip is achieved, as this is harder to notice in a colorless matrix. Although no method employing oral fluids has been published for endogenous compounds, Mercolini et al. have already developed a method for the quantification of cathinone analogs in such matrices [54].

Besides the solid analytical data that can be obtained by this approach, what is of high importance is its potential in clinical applications. Thus, complementary to the analytical validation, most studies performed clinical validation of the proposed method in real samples by comparing the analysis results obtained by the microsampling strategy with those from fluid biological samples. Most of the methods found good correlation between the results obtained with microsampling approaches and those obtained with conventional ones. Andriguetti et al. [21], Mathew et al. [22], Wang et al. [25], and Scuderi et al. [26] found a relevant bias when employing VAMS to analyze creatinine in blood samples, necessitating the application of correction factors to achieve values consistent with the reference values in fluid samples. Of course, one critical issue that should be stressed here is that the reference values for biomolecules in that type of specimen do not yet exist. As these may differ from the ranges in plasma, a lot of effort is needed for their establishment, which would facilitate their reliable application in clinical studies without barriers. Apart from this, no matter how many advantages this approach brings, it is still essential to address the concerns of clinicians and health care professionals and ensure that they are well-informed about VAMS’ benefits and reliability to encourage its adoption as a viable alternative to traditional venipuncture.

## 6. Conclusions

VAMS is a reliable tool for the analysis of endogenous analytes in biological samples. As discussed above, this approach is not always able to deliver superior results compared with the alternatives in terms of efficiency, stability, and accuracy. In addition, agreement with established reference values in venous blood remains a challenge. Therefore, before employing this technology in large-scale studies, preliminary analysis must be performed to ensure that the device fits for that specific application. Although conventional sampling techniques will still be necessary in disease diagnosis, this technology holds great promise for widespread implementation in clinical practice towards more safe, economic, and comfortable patient-centric diagnostic platforms that could transform the landscape in biomedical applications.

## Figures and Tables

**Table 2:** Pros and cons of the VAMS, DBS, and venipuncture collection techniques.

Technique	Pros	Cons
VAMS	Sampling of small volume of biological fluids (10, 20, and 30 μL) Volumetric collection of sample Enhanced stability at room temperature and no HCT effect (needs to be assessed in the study) Decreased costs for transport and storage Applicable for different biological matrices Enhanced patient comfort No need of specialized staff Reduced number of subjects needed in animal studies Possibility of automation Reduced biohazard risk	Higher costs of the device Generation of plastic waste Small sample volume might reduce sensitivity Necessitates expensive analytical instrumentation with high sensitivity
DBS	Sampling of small volume of biological fluids Enhanced stability at room temperature (must to be confirmed) Decreased costs for transport and storage Applicable for different biological matrices (for example, dried urine spots) Enhanced patient comfort Reduced number of subjects needed in animal studies Cheap Less generation of plastic waste Reduced biohazard risk	No volumetric collection of sample HCT effect Small sample volume might reduce sensitivity
Venipuncture	Cheaper devices Collection of large sample volumes does not require sensitive instrumentation	Decreased stability when compared with dried matrices Need for specialized staff for sample collection Patient discomfort Increased costs for transport and storage Generation of plastic waste Higher number of subjects needed in animal studies

## Supplementary Materials

The following supporting information can be downloaded at: https://www.mdpi.com/article/10.3390/metabo13101038/s1. Table S1: Analytical validation data of the developed methods for determination of creatinine in VAMS; Table S2: Analytical validation data of the developed methods for the determination of endogenous steroids in VAMs; Table S3: Analytical validation data of the developed method for determination of vitamins in VAMS; Table S4: Analytical validation data of the developed method for determination of phosphatidylethanols in VAMS; Table S5: Analytical validation data of the developed method for determination of phenylalanine, tyrosine, and homogentisic acid in VAMS; Table S6: Analytical validation data of the developed method for determination of amino acids and organic acids in VAMS; Table S7: Analytical validation data of the developed method for tryptophan pathway metabolites determination in VAMS.

## References

[1] Nys G., Kok M.G.M., Servais A.-C., Fillet M. (2017). Beyond Dried Blood Spot: Current Microsampling Techniques in the Context of Biomedical Applications. TrAC Trends Anal. Chem..

[2] Protti M., Mandrioli R., Mercolini L. (2019). Tutorial: Volumetric Absorptive Microsampling (VAMS). Anal. Chim. Acta.

[3] Londhe V., Rajadhyaksha M. (2020). Opportunities and Obstacles for Microsampling Techniques in Bioanalysis: Special Focus on DBS and VAMS. J. Pharm. Biomed. Anal..

[4] Li W., Lee M.S. (2014). Dried Blood Spots: Applications and Techniques.

[5] Kok M.G.M., Fillet M. (2018). Volumetric Absorptive Microsampling: Current Advances and Applications. J. Pharm. Biomed. Anal..

[6] Dodeja P., Giannoutsos S., Caritis S., Venkataramanan R. (2023). Applications of Volumetric Absorptive Microsampling Technique: A Systematic Critical Review. Ther. Drug Monit..

[7] Denniff P., Spooner N. (2010). The effect of hematocrit on assay bias when using DBS samples for the quantitative bioanalysis of drugs. Bioanalysis.

[8] Nugraha R.V., Yunivita V., Santoso P., Hasanah A.N., Aarnoutse R.E., Ruslami R. (2023). Analytical and Clinical Validation of Assays for Volumetric Absorptive Microsampling (VAMS) of Drugs in Different Blood Matrices: A Literature Review. Molecules.

[9] Mitra|VAMS Microsampling. https://www.neoteryx.com/volumetrically-accurate-micro-sampling-vams-collection-devices.

[10] Protti M., Cirrincione M., Mandrioli R., Rudge J., Regazzoni L., Valsecchi V., Volpi C., Mercolini L. (2022). Volumetric Absorptive Microsampling (VAMS) for Targeted LC-MS/MS Determination of Tryptophan-Related Biomarkers. Molecules.

[11] Denniff P., Spooner N. (2014). Volumetric Absorptive Microsampling: A Dried Sample Collection Technique for Quantitative bioanalysis. Anal. Chem..

[12] Denniff P., Parry S., Dopson W., Spooner N. (2015). Quantitative Bioanalysis of Paracetamol in Rats Using Volumetric Absorptive Microsampling (VAMS). J. Pharm. Biomed. Anal..

[13] Youhnovski N., Mayrand-Provencher L., Bérubé E.-R., Plomley J., Montpetit H., Furtado M., Keyhani A. (2017). Volumetric Absorptive Microsampling Combined with Impact-Assisted Extraction for Hematocrit Effect Free Assays. Bioanalysis.

[14] Kip A.E., Kiers K.C., Rosing H., Schellens J.H.M., Beijnen J.H., Dorlo T.P.C. (2017). Volumetric Absorptive Microsampling (VAMS) as an Alternative to Conventional Dried Blood Spots in the Quantification of Miltefosine in Dried Blood Samples. J. Pharm. Biomed. Anal..

[15] Parker S.L., Roberts J.A., Lipman J., Wallis S.C. (2015). Quantitative Bioanalytical Validation of Fosfomycin in Human Whole Blood with Volumetric Absorptive Microsampling. Bioanalysis.

[16] De Kesel P.M.M., Lambert W.E., Stove C.P. (2015). Does Volumetric Absorptive Microsampling Eliminate the Hematocrit Bias for Caffeine and Paraxanthine in Dried Blood Samples? A Comparative Study. Anal. Chim. Acta.

[17] Miao Z., Farnham J.G., Hanson G., Podoll T., Reid M.J. (2015). Bioanalysis of Emixustat (ACU-4429) in Whole Blood Collected with Volumetric Absorptive Microsampling by LC-MS/MS. Bioanalysis.

[18] Ye Z., Gao H. (2017). Evaluation of Sample Extraction Methods for Minimizing Hematocrit Effect on Whole Blood Analysis with Volumetric Absorptive Microsampling. Bioanalysis.

[19] Mano Y., Kita K., Kusano K. (2015). Hematocrit-Independent Recovery Is a Key for Bioanalysis Using Volumetric Absorptive Microsampling Devices, MitraTM. Bioanalysis.

[20] John H., Willoh S., Hörmann P., Siegert M., Vondran A., Thiermann H. (2016). Procedures for Analysis of Dried Plasma Using Microsampling Devices to Detect Sulfur Mustard-Albumin Adducts for Verification of Poisoning. Anal. Chem..

[21] Andriguetti N.B., Lisboa L.L., Hahn S.R., Pagnussat L.R., Antunes M.V., Linden R. (2019). Simultaneous Determination of Vancomycin and Creatinine in Plasma Applied to Volumetric Absorptive Microsampling Devices Using Liquid Chromatography-Tandem Mass Spectrometry. J. Pharm. Biomed. Anal..

[22] Mathew B.S., Mathew S.K., Aruldhas B.W., Prabha R., Gangadharan N., David V.G., Varughese S., John G.T. (2022). Analytical and Clinical Validation of Dried Blood Spot and Volumetric Absorptive Microsampling for Measurement of Tacrolimus and Creatinine after Renal Transplantation. Clin. Biochem..

[23] Marshall D.J., Kim J.J., Brand S., Bryne C., Keevil B.G. (2020). Assessment of Tacrolimus and Creatinine Concentration Collected Using Mitra Microsampling Devices. Ann. Clin. Biochem..

[24] Zwart T.C., Metscher E., van der Boog P.J.M., Swen J.J., de Fijter J.W., Guchelaar H.-J., de Vries A.P.J., Moes D.J.A.R. (2022). Volumetric Microsampling for Simultaneous Remote Immunosuppressant and Kidney Function Monitoring in Outpatient Kidney Transplant Recipients. Br. J. Clin. Pharmacol..

[25] Wang X., Dai X., Wan S., Fan Y., Wu L., Xu H., Yan L., Gong X., Li Y., Luo Y. (2022). A Volumetric Absorptive Microsampling UPLC-MS/MS Method for Simultaneous Quantification of Tacrolimus, Mycophenolic Acid and Creatinine in Whole Blood of Renal Transplant Recipients. Pharmaceutics.

[26] Scuderi C.E., Parker S.L., Jacks M., John G.T., McWhinney B., Ungerer J., Mallett A.J., Healy H.G., Roberts J.A., Staatz C.E. (2023). Serum Creatinine and Tacrolimus Assessment With VAMS Finger-Prick Microsampling: A Diagnostic Test Study. Kidney Med..

[27] Chang W.C.-W., Cowan D.A., Walker C.J., Wojek N., Brailsford A.D. (2020). Determination of Anabolic Steroids in Dried Blood Using Microsampling and Gas Chromatography-Tandem Mass Spectrometry: Application to a Testosterone Gel Administration Study. J. Chromatogr. A.

[28] Protti M., Marasca C., Cirrincione M., Sberna A.E., Mandrioli R., Mercolini L. (2020). Dried Urine Microsampling Coupled to Liquid Chromatography-Tandem Mass Spectrometry (LC-MS/MS) for the Analysis of Unconjugated Anabolic Androgenic Steroids. Molecules.

[29] Heussner K., Rauh M., Cordasic N., Menendez-Castro C., Huebner H., Ruebner M., Schmidt M., Hartner A., Rascher W., Fahlbusch F.B. (2017). Adhesive Blood Microsampling Systems for Steroid Measurement via LC-MS/MS in the Rat. Steroids.

[30] Marshall D.J., Adaway J.E., Hawley J.M., Keevil B.G. (2020). Quantification of Testosterone, Androstenedione and 17-Hydroxyprogesterone in Whole Blood Collected Using Mitra Microsampling Devices. Ann. Clin. Biochem..

[31] Tuma C., Thomas A., Braun H., Thevis M. (2023). Quantification of 25-Hydroxyvitamin D2 and D3 in Mitra^®^ Devices with Volumetric Absorptive Microsampling Technology (VAMS^®^) by UHPLC-HRMS for Regular Vitamin D Status Monitoring. J. Pharm. Biomed. Anal..

[32] Verstraete J., Stove C. (2021). Patient-Centric Assessment of Thiamine Status in Dried Blood Volumetric Absorptive Microsamples Using LC-MS/MS Analysis. Anal. Chem..

[33] Verstraete J., Stove C. (2021). Volumetric Absorptive Microsampling (VAMS) as a Reliable Tool to Assess Thiamine Status in Dried Blood Microsamples: A Comparative Study. Am. J. Clin. Nutr..

[34] Van Uytfanghe K., Ramirez Fernandez M.D.M., De Vos A., Wille S.M., Stove C.P. (2021). Quantitation of Phosphatidylethanol in Dried Blood after Volumetric Absorptive Microsampling. Talanta.

[35] Carling R.S., Emmett E.C., Moat S.J. (2022). Evaluation of Volumetric Blood Collection Devices for the Measurement of Phenylalanine and Tyrosine to Monitor Patients with Phenylketonuria. Clin. Chim. Acta.

[36] Gao L., Smith N., Kaushik D., Milner S., Kong R. (2023). Validation and Application of Volumetric Absorptive Microsampling (VAMS) Dried Blood Method for Phenylalanine Measurement in Patients with Phenylketonuria. Clin. Biochem..

[37] Taylor J.M., Hughes A.T., Milan A.M., Rudge J., Davison A.S., Ranganath L.R. (2018). Evaluation of the Mitra Microsampling Device for Use with Key Urinary Metabolites in Patients with Alkaptonuria. Bioanalysis.

[38] Kok M.G.M., Nix C., Nys G., Fillet M. (2019). Targeted Metabolomics of Whole Blood Using Volumetric Absorptive Microsampling. Talanta.

[39] Kapadnis U., Locuson C., Okamura H., Rienzo G.D., Cotter C., Zhu D., Narayanaswami R., Castro-Perez J., Marathe P., Yang W.-C. (2023). Volumetric Absorptive Microsampling as an Effective Microsampling Technique for LC-MS/MS Bioanalysis of Biomarkers in Drug Discovery. Bioanalysis.

[40] Gowda S., Desai P.B., Kulkarni S.S., Hull V.V., Math A.A.K., Vernekar S.N. (2010). Markers of Renal Function Tests. N. Am. J. Med. Sci..

[41] World Anti-Doping Agency (2013). Prohibited List. https://www.wada-ama.org/en/prohibited-list.

[42] World Anti-Doping Agency (2009). World Anti-Doping Code. https://www.wada-ama.org/en/resources/world-anti-doping-program/world-anti-doping-code.

[43] World Anti-Doping Code (2021). Athlete Biological Passport Operating Guidelines and Compilation of Required Elements, Version 8.0. http://www.wada-ama.org.

[44] Fahlbusch F.B., Ruebner M., Rascher W., Rauh M. (2013). Combined Quantification of Corticotropin-Releasing Hormone, Cortisol-to-Cortisone Ratio and Progesterone by Liquid Chromatography-Tandem Mass Spectrometry in Placental Tissue. Steroids.

[45] Millward D., Root A.D., Dubois J., Cohen R.P., Valdivia L., Helming B., Kokoskie J., Waterbrook A.L., Paul S. (2020). Association of Serum Vitamin D Levels and Stress Fractures in Collegiate Athletes. Orthop. J. Sports Med..

[46] Bouillon R., Manousaki D., Rosen C., Trajanoska K., Rivadeneira F., Richards J.B. (2022). The Health Effects of Vitamin D Supplementation: Evidence from Human Studies. Nat. Rev. Endocrinol..

[47] Thacher T.D., Clarke B.L. (2011). Vitamin D Insufficiency. Mayo Clin. Proc..

[48] Larkin E.K., Gebretsadik T., Koestner N., Newman M.S., Liu Z., Carroll K.N., Minton P., Woodward K., Hartert T.V. (2011). Agreement of Blood Spot Card Measurements of Vitamin D Levels with Serum, Whole Blood Specimen Types and a Dietary Recall Instrument. PLoS ONE.

[49] Schröck A., Henzi A., Bütikofer P., König S., Weinmann W. (2018). Determination of the formation rate of phosphatidylethanol by phospholipase D (PLD) in blood and test of two selective PLD inhibitors. Alcohol.

[50] Luginbühl M., Young R.S.E., Stoeth F., Weinmann W., Blanksby S.J., Gaugler S. (2021). Variation in the Relative Isomer Abundance of Synthetic and Biologically Derived Phosphatidylethanols and Its Consequences for Reliable Quantification. J. Anal. Toxicol..

[51] van Spronsen F.J., van Wegberg A.M., Ahring K., Bélanger-Quintana A., Blau N., Bosch A.M., Burlina A., Campistol J., Feillet F., Giżewska M. (2017). Key European Guidelines for the Diagnosis and Management of Patients with Phenylketonuria. Lancet Diabetes Endocrinol..

[52] Microsampling in Pre-Clinical Research. https://www.neoteryx.com/blood-microsampling-animal-research?hsCtaTracking=1dc2b846-de1c-4466-a912-bbed54938101%7Cd1f9bef1-9898-424a-a687-b9f2c0d08705.

[53] Thangavelu M.U., Wouters B., Kindt A., Reiss I.K.M., Hankemeier T. (2023). Blood Microsampling Technologies: Innovations and Applications in 2022. Anal. Sci. Adv..

[54] Mercolini L., Protti M., Catapano M.C., Rudge J., Sberna A.E. (2016). LC-MS/MS and volumetric absorptive microsampling for quantitative bioanalysis of cathinone analogues in dried urine, plasma and oral fluid samples. J. Pharm. Biomed. Anal..

