# Supplementary material for: Volumetric Absorptive Microsampling in the Analysis of Endogenous Metabolites
Source: Metabolites. 2023 Sep 26;13(10):1038. doi: 10.3390/metabo13101038 (PMC10609074; doi:10.3390/metabo13101038)
Supplement: Supplementary file 1 [file metabolites-13-01038-s001.zip › Table S1.pdf]

Table S1. Analytical validations of the developed methods for determination of creatinine in VAMS

Analytes	Linearity (R^2)	LOQ	Intra-Day Precision CV%)	Inter-Day Precision (CV%)	Accuracy (% bias)	Matrix Effects (%)	Recovery (%)	Hematocrit effects	Stability	Additional data	Ref
Cr	0.9941	5 mg/L	3.4% (5 µg/mL)<br>7.5% (7.5 µg/mL)<br>4% (35 µg/mL)<br>4.4% (75 µg/mL)	4.5% (5 µg/mL)<br>5.6% (7.5 µg/mL)<br>3.4% (35 µg/mL)<br>6.9% (75 µg/mL)	99.1% (5 µg/mL)<br>102.9% (7.5 µg/mL)<br>98% (35 µg/mL)<br>98.6% (75 µg/mL)	3.74% (7.5 µg/mL)<br>1.02 (75 µg/mL)	77.8% (7.5 µg/mL)<br>87.5% (75 µg/mL)	_	Stable for 14 days (96.4–99.7% of recovery)	_	[21]
Cr	>0.99	0.2 mg/dL	8.4% (0.2 mg/dL)<br>5.5% (1mg/dL)<br>3.6% (4mg/dL)<br>2.2% (5mg/dL)	3% (0.2 mg/dL)<br>9.9% (1mg/dL)<br>3.1% (4mg/dL)<br>-1.6% (5mg/dL)	108.2% (1mg/dL)<br>102.3% (4mg/dL)	-3.4% (1mg/dL)<br>-4.7% (4mg/dL)	112.8% (1mg/dL)<br>107.4% (4mg/dL)	Max. Bias of −10.3% (HCT 0.55 $L.L^{-1}$ and 0.25 $L.L^{-1}$)	-7.2% over 5 days at 3°C and 14.8% for 30 days at -80°C	_	[22]
Cr	0.998	16 µmol/mL	1.8% (16 µmol/mL PBS based)<br>2.3% (150 µmol/mL PBS based)<br>1.1% (600 µmol/mL PBS based)	6.5% (115 µmol/mL)<br>5.6% (184 µmol/mL)<br>7.2% (289 µmol/mL)	98% against External Quality Assurance Standards; 94.5% against venous blood analysis	<3%	100% - 115% (mean recovery 108%)	−1.1% to 9.6%; mean 4.3% bias	<10% at 4°C. -20°C. and rt. Stable at 37°C for 24 hours.	_	[23]
Cr	0.99	3 mg/L	2.5% (6050 µg/L)<br>3.9% (11050 µg/L)<br>2.5% (21050 µg/L)	7.75% (6050 µg/L)<br>12.36% (11050 µg/L)<br>19.53% (21050 µg/L)	92.4% (6050 µg/L)<br>106.6% (11050 µg/L)<br>100.1% (21050 µg/L)	_	_	No HCT dependencies.	Stability only performed for DBS samples	_	[24]
Cr	> 0.99	10 nmol/mL	5.33% (10 nmol/mL)<br>5.21% (30 nmol/mL)<br>5.3% (400 NMOL/mL)<br>2.82% (800 nmol/ML)	5.42% (10 nmol/mL)<br>5.64% (30 nmol/mL)<br>4.52% (400 NMOL/mL)<br>3.29% (800 nmol/ML)	−5.45% - 4.9%	+0.96% to +1.15% (30 nmol/mL)<br><br>+ 0.98% to +1.08% (800 nmol/mL)	101.75% (30 nmol/mL)<br>95.09 (400 nmol/mL)<br>96.26 (800 nmol/mL)	−4.65% (30 nmol/mL)<br>+1.75% (800 nmol/mL)	Stable for 1 month under 4 and 23 ºC (SD<15%)	_	[25]

Abbreviations: creatinine (Cr), room temperature (rt)
