# Supplementary material for: Volumetric Absorptive Microsampling in the Analysis of Endogenous Metabolites
Source: Metabolites. 2023 Sep 26;13(10):1038. doi: 10.3390/metabo13101038 (PMC10609074; doi:10.3390/metabo13101038)
Supplement: Supplementary file 1 [file metabolites-13-01038-s001.zip › Table S2.pdf]

Table S2. Analytical validation data of the developed methods for the determination of endogenous steroids in VAMs.

Analytes	Linearity (R^2)	LOQ	Intra-Day Precision (CV%)	Inter-Day Precision (CV%)	Accuracy	Matrix Effect (%)	Recovery (%)	Hematocrit Effects	Stability	Ref
T	0.9995	0.78 ng/mL	3.6% (2 ng/mL)<br>5.2% (10 ng/mL)<br>10.4% (25 ng/mL)	8.1% (2 ng/mL)<br>4.5% (10 ng/mL)<br>11.6% (25 ng/mL)	1.6% (2 ng/mL)<br>0.9% (10 ng/mL)<br>3.8% (25 ng/mL)	116%	89%	Not evaluated.	> 80% at RT after two months	[27]
T; ET; DHT; DHEA	> 0.99	T and ET: 1.0 ng/mL; DHEA and DHT: 1.5 ng/mL	T: 7% (LOQ)<br>6.4% (375 ng/ml)<br>5.9% (750 ng/ml)<br>ET: 6.8% (LOQ)<br>6.2% (375 ng/ml)<br>5.8 % (750 ng/ml)<br>DHT: 7% (LOQ)<br>6.9% (375 ng/ml)<br>6.7% (750 ng/ml)<br>DHEA: 7%(LOQ)	T: 6.6% (LOQ)<br>5.9% (375 ng/ml)<br>5.3% (750 ng/ml)<br>ET: 6.3% (LOQ)<br>5.8% (375 ng/ml)<br>5.5 % (750 ng/ml)<br>DHT: 6.9% (LOQ)<br>6.6% (375 ng/ml)<br>6.3% (750 ng/ml)<br>DHEA:6.9% (LOQ),<br>6.4 %(375 ng/ml),<br>6.1% (375 ng/ml)	89-97%	T: 6.1-8.7 %<br>ET: 4.2-7%<br>DHT: 3.2-5.1%<br>DHEA:6.8-8.8 %	LOQ<br>T: 77.3 ± 3.3<br>ET:79.1± 4.6<br>DHT: 81.6 ± 2.6<br>DHEA: 90.1 ± 3.7<br>375 ng/ml<br>T: 78.4 ± 3.8<br>ET:79.1 ± 3.1<br>DHT: 80.4 ± 2.7<br>DHEA: 90.2 ± 3.4<br>750 ng/ml<br>T: 80.3 ± 2.4 ET: 82.0 ± 2.8<br>DHT: 82.7 ± 1.9<br>DHEA: 92.0 ± 2.7	HCT effects NA (urine specimen)	Stability Results after 1 year, at RT:<br>LOQ<br>T: 84% ET: 87<br>DHT: 84<br>DHEA: 83<br>375 ng/ml<br>T: 85 ET: 88<br>DHT:86<br>DHEA: 84<br>750 ng/ml<br>T: 85 ET: 88<br>DHT: 88<br>DHEA: 86	[28]
Cortisol (in Human);<br>Cortisone (in Human);<br>Corticosterone (in Rat)<br>11-Dehydrocorticosterone	0.993 - 0.999	-	Cortisol: 2%<br>Cortisone: 32%<br>Corticosterone: 18%<br>11-Dehydrocorticosterone: 29%	-	-	-	cortisol-d4: >96%<br>corticosterone-d8: >80%	HCT effect present: HCT-adapted volume factors are mandatory.	-	[29]
T; A4;<br>17-OHP	> 0.999	T: 1 nmol/L<br>A4: 1nmol/L<br>17-OHP: 4nmol/L	T:<br>3.2 % (3.3 nmol/L)<br>3.0 % (12.5 nmol/L)<br>2.2 % (24.1 nmol/L)<br>A4:<br>3.8 % (3.0 nmol/L)<br>2.7 % (10.7 nmol/L)<br>2.4 % (19.7 nmol/L)<br>17-OHP:<br>6.5 % (4.8 nmol/L)<br>3.1 % (20.8 nmol/L)<br>3.5 % (38.1 nmol/L)	T:<br>5.3 % (3.3 nmol/L)<br>4.5% (12.5 nmol/L)<br>5.7% (24.1 nmol/L)<br>A4:<br>7.6% (3.0 nmol/L)<br>7.6 % (10.7 nmol/L)<br>5.8% (19.7 nmol/L)<br>17-OHP:<br>7.5% (4.8 nmol/L)<br>4.4% (20.8 nmol/L)<br>7.2% (38.1 nmol/L)	T:<br>-0.6 % (3.3 nmol/L)<br>1.3 % (12.5 nmol/L)<br>1.8 % (24.1 nmol/L)<br>A4:<br>-5.4 % (3.0 nmol/L)<br>-8.8 % (10.7 nmol/L)<br>-5.7 % (19.7 nmol/L)<br>17-OHP:<br>-7.1 % (4.8 nmol/L)<br>6.7 % (20.8 nmol/L)<br>12.5 % (38.1 nmol/L)	"The imprecision (%CV) and bias were determined to be <10%"	101 - 104% (T)<br>95 - 100% (A4)<br>96 - 98% (17-OHP)	–12.8% - 4.3%	14 days at RT,<br>20 days at -20 ºC,<br>Storage at 60ºC not acceptable<br>(Acceptance criteria: ± 15.0%)	[30]

Abbreviations: Testosterone (T), Androstenedione (A4), 17-hydroxyprogesterone (17-OHP), Epitestosteorne (ET), Dihydrotestoterone (DHT), Dehydroepiandrosterone (DHEA)
