# Supplementary material for: Volumetric Absorptive Microsampling in the Analysis of Endogenous Metabolites
Source: Metabolites. 2023 Sep 26;13(10):1038. doi: 10.3390/metabo13101038 (PMC10609074; doi:10.3390/metabo13101038)
Supplement: Supplementary file 1 [file metabolites-13-01038-s001.zip › Table S3.pdf]

Table S3. Analytical validation of the developed method for determination of vitamins in VAMS

**Analytes**	**Linearity (R^2)**	**LOQ**	**Intra-Day Precision CV%)**	**Inter-Day Precision (CV%)**	**Accuracy (% and CI)**	**Matrix Effects (%)**	**Recovery (%)**	**Hematocrit effects**	**Stability**	**Additional data**	**Ref**
25(OH)D ; 25(OH)D3	0 - 150 ng/ml, r2 > 0.99	5 ng/ml	25(OH)D2: <10% (15.1 ng/ml and 49.1 ng/ml)<br><br>25(OH)D3: < 10% (14.5 ng/ml and 53.6 ng/ml)	25(OH)D2: 7% - 11% (15.1 ng/ml and 49.1 ng/ml)<br><br>25(OH)D3: 7% – 11% (14.5 ng/ml and 53.6 ng/ml)	25(OH)D2: 98%-110% (15.1 ng/ml and 49.1 ng/ml)<br><br>25(OH)D3: 98%-110% (15.1 ng/ml and 49.1 ng/ml)	25(OH)D2: 81%<br><br>25(OH)D3: 70%	25(OH)D2: > 68%<br><br>25(OH)D3: > 68%	–	**25(OH)D2**<br>VAMs samples dried for 2 hrs and stored in a ziplock with desiccant:<br>• 28 days at -18°C (< 3% degradation.<br>• 3 days at rt (20°C) (85%).<br>• 28 days at rt (20°C) (38%).<br><br>Storage in original Mitra cartridge device.<br>• 28 days at rt (20°C) (< 8% degradation)<br><br>**25(OH)D3**<br>VAMs samples dried for 2 hrs and stored in ziplock with desiccant:<br>• 28 days at -18°C (< 3% degradation.<br>• 3 days at rt (20°C) (93%).<br>• 28 days at rt (20°C) (50%).<br><br>Storage in original Mitra cartridge device.<br>• 28 days at rt (20°C) (< 3% degradation).	**Selectivity** 25(OH)D2 and 25(OH)D3: No interfering peaks at predicted retention times.<br><br>**Carryover**<br>25(OH)D2 and 25(OH)D3: < 0.01%<br><br>**Robustness**<br>25(OH)D2 at (15.1 ng/ml and 49.1 ng/ml) and 25(OH)D3 at (14.5 ng/ml and 53.6 ng/ml) analyzed under the following conditions:<br>• Time in ultrasound (20, 30, and 45 mins).<br>• Temperature in vacuum centrifuge (45°C and 60°C).<br><br>No effect on concentration	[31]

TDP	9.3 ng/ml - 473 ng/ml)	9.3 ng/ml	**VLB**<br>2.5% (9.3 ng/ml)<br>4% (19 ng/ml)<br>4.6% (62.2 ng/ml)<br>5.7% (341 ng/ml)<br>Native blood: 5.6%<br><br>**VAMS**<br>3.9% (9.3 ng/ml)<br>2.8% (19 ng/ml)<br>5% (62.2 ng/ml)<br>5.2% (341 ng/ml)<br>Native blood: 3.1%	**VLB**<br>5.5% (9.3 ng/ml)<br>5% (19 ng/ml)<br>4.6% (62.2 ng/ml)<br>5.7% (341 ng/ml)<br>Native blood: 7.6%<br><br>**VAMS**<br>12.4% (9.3 ng/ml)<br>5.2% (19 ng/ml)<br>5% (62.2 ng/ml)<br>5.2% (341 ng/ml)<br>Native blood: 4.8%	**VLB**<br>0.7% (9.3 ng/ml)<br>-5.4% (19 ng/ml)<br>3.1% (62.2 ng/ml)<br>0.3% (341 ng/ml)<br>Native blood: NA<br><br>**VAMS**<br>5.6% (9.3 ng/ml)<br>-6.2% (19 ng/ml)<br>-0.1% (62.2 ng/ml)<br>1.7% (341 ng/ml)<br>Native blood: NA	**VLB NON-IS**<br>0.2 HCT: 9% (18% CV).<br>0.6 HCT: 7% (18% CV)<br><br>**VLB with IS**<br>0.2 HCT:109% (4%CV)<br>0.6 HCT: 99% (5%CV)<br><br>**VAMS NON- IS**<br>0.2 HCT: 15% (19% CV)<br>0.6 HCT: 13% (22% CV)<br><br>**VAMS with IS**<br>0.2 HCT: 98% (13% CV)<br>0.6 HCT: 99% (5% CV)	**VLB**<br>107% +/- 14% SD (50 ng/ml)<br>103% +/- 4% SD (300 ng/ml)<br><br>**VAMS**<br>92% +/- 6% SD (50 ng/ml)<br>96% +/- 7% SD (300 ng/ml)	See Matrix effects column.	**VLB**<br>12 hrs at 37°C. 24 hrs at rt (store at -80 °C prior to analysis to enable extraction of TDP).<br><br>**VAMS**<br>7 days at 60 °C (no light protection). 7 days at 28 °C and 80% humidity with desiccant (no light protection). 24 hrs at rt and 80% humidity without desiccant (no light protection). > 7 days at rt for at least 1 month.<br><br>**Extra notes**<br>Processed samples stable for 48 hrs at 8 °C in the autosampler; can be reanalyzed after 2 weeks at - 20 °C or - 80 °C.<br>For **VLB** and **VAMS** there was no difference in light vs light protected storage.	**Selectivity**<br>Ion ratios between neat standards and native blood were within +/- 20% acceptance criteria. No unacceptable interferences.<br><br>**Carryover**<br>No unacceptable carryover (< 20% LOQ) in blanks analyzed after 89 ng/ml blood samples.<br><br>**Method comparison (Bland Altman)**<br>**VLB vs VAMS**<br>2.4% bias within 95% CI (-1.2 to 5.94).<br><br>VLB: 5%CV<br>VAMS: 6% CV	[32]
TDP	9.30 ng/ml - 473 ng/ml	9.30 ng/ml	**CVAMS vs VVAMS**: 6.5% CV vs 4.0% CV ($p < 0.05$).<br><br>**VVAMS vs VLB:** 4.4% CV vs 2.2% ($p < 0.05$).<br><br>**CVAMS vs VLB:** 6.5% CV vs 2.2% CV.<br><br>2/3 of samples analyzed were within criteria (< 20% CV).		**CVAMS vs VVAMS:** -0.4% bias. Deming regression slope and intercept within 95% CI.<br><br>**VVAMS vs VLB:** 0.7% bias. Deming regression slope and intercept within 95% CI.<br><br>**CVAMS vs VLB:** -1% bias. Deming regression slope and intercept within 95% CI.	-	-	**CVAMS vs VLB:** No impact of HCT ($p > 0.05$); slope not significantly different from 0.	**CVAMS** transported in non-controlled condition (no time or temperature control) in envelope with desiccant vs **controlled condition** (maximum 10 hrs, cool temperatures, and protected from light):<br><br>1% mean bias, slope of trend lines for comparison not significantly different from 0. No significant difference in transportation means ($p > 0.05$).	-	[33]

Abbreviations: 25 hydroxyvitamin D2 (25(OH)D2), room temperature (rt), 25 hydroxyvitamin D3 (25(OH)D3), Thiamine diphosphate (TDP), confidence interval (CI), coefficient of variation (CV), probability (p), capillary VAMS (CVAMS), Venous VAMS (VVAMS), venous liquid blood (VLB), venous liquid blood (VLB), internal standard (IS), confidence interval (CI), hematocrit (HCT), matrix effects (ME), standard deviation (SD).
