# Supplementary material for: Volumetric Absorptive Microsampling in the Analysis of Endogenous Metabolites
Source: Metabolites. 2023 Sep 26;13(10):1038. doi: 10.3390/metabo13101038 (PMC10609074; doi:10.3390/metabo13101038)
Supplement: Supplementary file 1 [file metabolites-13-01038-s001.zip › Table S4.pdf]

Table S4. Analytical validation of the developed method for determination of phosphatidylethanol in VAMS

Analytes	Linearity (R^2)	LOQ	Intra-Day Precision CV%)	Inter-Day Precision (CV%)	Accuracy (% bias)	Matrix Effects (%)	Recovery (%)	Hematocrit effects	Stability	Ref
PEth (UGent laboratory)	-	1.7 ng/ml (LOD)<br>10 ng/ml (LLOQ)<br>2000 ng/ml (ULOQ)	7.8% (10 ng/ml)<br>6.2% (30 ng/ml)<br>7.9% (500 ng/ml)<br>5.1% (1500 ng/ml)<br>6.9% (735 ng/ml)<br>7.9% (244 ng/ml)<br>8.2% (465 ng/ml)<br>8.6% (146 ng/ml)<br>9.6% (15.7 ng/ml)	10.9% (10 ng/ml)<br>4.8% (30 ng/ml<br>7.8% (500 ng/ml)<br>10.1% (1500 ng/ml)<br>4.8% (735 ng/ml)<br>7.5% (244 ng/ml)<br>7.1% (465 ng/ml)<br>10.8% (146 ng/ml)<br>6.6% (15.7 ng/ml)	5.2% (10 ng/ml)<br>-4.5% (30 ng/ml)<br>-5.5% (500 ng/ml)<br>-6.5% (1500 ng/ml)	**Non-IS**<br>79.6 % (21% RSD) (low QC)<br>77.8% (16% RSD) (high QC)<br><br>**IS**<br>100% (7.9% RSD) (low QC)<br>95.9% (5.2% RSD) (high QC)	**Non-IS**<br>64.2% (12% RSD) (low QC low HCT)<br>47.6% (14% RSD) (low QC normal HCT)<br>43.9 (14% RSD) (low QC high HCT)<br><br>62.5% (14% RSD) (high QC low HCT)<br>64.1% (14% RSD) (high QC normal HCT)<br>61.3% (12% RSD) high QC high HCT<br><br>**IS**<br>61.0% (10% RSD) (low QC low HCT)<br>46.6% (9.5% RSD) (low QC normal HCT)<br>42.5% (13% RSD) (low QC high HCT)<br><br>54.5% (15% RSD) (high QC low HCT)<br>54.0% (8.1% RSD) (high QC normal HCT)<br>58.6% (10% RSD) (high QC high HCT)	See recovery column.	One week stability at 4°C, RT, and 45° C. One month stability at RT.	[34]
Peth (NICC laboratory)	-	10 ng/ml (LLOQ)<br>2000 ng/ml (ULOQ)	15.4% (10 ng/ml)<br>6.2% (30 ng/ml)<br>9.8% (500 ng/ml)<br>7.1% (1500 ng/ml)	13.3% (10 ng/ml)<br>7% (30 ng/ml)<br>4.6% (500 ng/ml)<br>5% (1500 ng/ml)	4.4 (10 ng/ml)<br>-10.3% (30 ng/ml)<br>-8.9% (500 ng/ml)<br>8.5% (1500 ng/ml)	**Non-IS**<br>119.5% (24% RSD) (low QC)<br>107.8% (14% RSD) (high QC)<br><br>**IS**<br>110.7% (5% RSD) (low QC) 108% (6% RSD) (high QC)	**LLE efficiency**<br>**Non-IS**<br>71.1% (12% RSD) (low QC low HCT)<br>70.6% (11% RSD)(low QC normal HCT)<br>79.4% (14% RSD) (low QC high HCT)<br>70.8% (14% RSD) (high QC low HCT)<br>78.1 (12% RSD) (high QC normal HCT)<br>81.1 (6% RSD) (high QC high HCT)	See recovery column.	One week stability at 4°C, RT, and 45° C. One month stability at RT.	[34]

Abbreviations: phosphatidylethanol (PEth), Ghent University Laboratory of toxicology (UGent); National Institute of Criminalistics and Criminology (NICC), low limit of quantitation (LLOQ), upper limit of quantitation (ULOQ); hematocrit effects (HCT), internal standards (IS), liquid liquid extraction (LLE), relative standard deviation (RSD).
