# Supplementary material for: Volumetric Absorptive Microsampling in the Analysis of Endogenous Metabolites
Source: Metabolites. 2023 Sep 26;13(10):1038. doi: 10.3390/metabo13101038 (PMC10609074; doi:10.3390/metabo13101038)
Supplement: Supplementary file 1 [file metabolites-13-01038-s001.zip › Table S5.pdf]

Table S5. Analytical validation of the developed method for determination of phenylalanine, tyrosine, and homogentisic acid in VAMS.

Analytes	Linearity (R^2)	LOQ	Intra-Day Precision CV%)	Inter-Day Precision (CV%)	Accuracy (% bias)	Matrix Effects (%)	Recovery (%)	Hematocrit effects	Stability	Additional data	Ref
Phe and Tyr	_	_	Phe:<br>5.6% CV (endogenous, 508, and 811) µmol/L<br><br>Tyr:<br>6.2% CV (endogenous, 349, and 557) µmol/L		Phe:<br>0.1% bias (endogenous)<br>-15.8% bias (508 µmol/L)<br>-20.4% bias (811 µmol/L)<br><br>Tyr:<br>-12% to -25% bias (349 – 557) µmol/L	_	_	Phe:<br>-12.8% bias (508 µmol/L; 44.4% and 63.3% HCT)<br><br>Tyr:<br>-17.3% bias (349 µmol/L; 44.4% and 63.3% HCT)	15 days at rt and 4°C. p>0.01.	**Effect of application of different volumes and contact time after tip is filled**<br><br>Phe:<br>No effect on concentrations, p > 0.01 (20, 50, and 200 µl; 508 µmol/L; 0, 2, 5, and 10 s)<br><br>Tyr:<br>No effect on concentrations, p > 0.01 (349 µmol/L; 0, 2, 5, and 10 s)	[35]
Phe and Phe-surrogate	30 - 3000 µmol/L, r^2 = 0.9894	30 µmol/L	Phe:<br>3.1% - 7.7% CV (75, 450, and 2250) µmol/L<br><br>Phe-surrogate:<br>3.5% - 9.3% CV (75, 450, and	Phe:<br>4.9% - 6.2 % CV (75, 450, and 2250) µmol/L<br><br>Phe-surrogate:<br>6.0% - 8.8% CV (75, 450, and	**Intra accuracy**<br>Phe:<br>-3.9% - 13.0% bias (75, 450, and 2250) µmol/L<br><br>Phe-surrogate:<br>-5.3% - 10.9% bias (75, 450, and 2250) µmol/L	Phe: no impact on accuracy and precision (75, 450, and 2250) µmol/L<br><br>Phe-surrogate: -13.5% to 4.9% bias.	Phe-surrogate: 88% to 108.4% (75, 450, and 2250) µmol/L	Phe: -10.7 to 3.1% bias; 2.5% to 9.3% CV (75 and 2250 µmol/L: 0.3, 0.5, and 0.7 HCT)	3.25 hrs. in WB at 37°C and room temperature. 232 days (in the VAMS clamshell) at rt.<br>15 days at 45°C.<br>200 hrs. in extract at rt. 208.75 hrs. for reinjection precision at rt.	**Lipemic effect**<br>Phe-surrogate: 0.4% to 3.6% bias; 1.9% to 7.9% CV.<br>**(**75 and 2250 µmol/L)<br><br>**Selectivity**<br>Phe and Phe-surrogate:	[36]

			2250) μmol/L	2250) μmol/L	**Inter accuracy**<br>Phe:<br>0.9% - 11.4% bias (75, 450, and 2250) μmol/L<br><br>Phe-surrogate:<br>-1.3% - 9.9% bias (75, 450, and 2250) μmol/L	1.2% to 12.6% CV (75 and 2250) μmol/L				No meaningful effect in quantification.<br><br>**Method comparison**<br>Phe VAMS vs plasma samples Pearson Correlation (Significant correlation. p < 2.2*10 e^6, R = 0.9756).<br>Passing Bablock (Intercept ~ 0; 95% CI contained 0, slope ~ 1, value range < LLOQ (30 μmol/L), % bias < 5%).<br>Bland Altman (Proportional mean difference = -0.04246; 95% CI (-0.05904 to -0.02589).	
Phe; Tyr; HGA in urine VAMS	_	_	**Sampling precision**<br>3.7% CV (absorb till tip is filled and wait 2 additional seconds)<br>4.1% CV (sample for 6 s)<br>4.6% CV (sample for 10 s)<br>1.2% CV (good pipetting)<br>4.6% CV (bad pipetting)		**Sampling accuracy measured vols (actual vol = 10 μL); <0.47 SD**<br>10.3 μL (absorb till tip is filled and wait 2 additional seconds)<br>10.4 μL (sample for 6 s)<br>10.6 μL (sample for 10 s)	_	Phe (< 9.3 SD):<br>83.1% (5 min agitation)<br>95.4% (15 min agitation)<br>99% (5 min no agitation)<br>101.1% (15 min no agitation)<br><br>Tyr (< 7.0 SD)<br>86.9% (5 min agitation)<br>104.3% (15 min agitation)	_	Phe:<br>Stable for up to 28 days (20°C day light/ no light; 4°C no light, 37°C no light).<br><br>Small increase in overall stability at 37°C for up to 28 days (AC vs non-AC).<br><br>Tyr:	**Mitra drying time:**<br>Constant Mitra tip weight reached after drying for 45 mins.<br><br>**Method comparison (urine VAMS vs liquid urine)**<br><br>Phe:	[37]

				10.1 μL (good pipetting)<br>10.2 μL (bad pipetting)		106.4% (5 min no agitation)<br>103.9% (15 min no agitation)<br><br>HGA (<11.9 SD)<br>82.2% (5 min agitation)<br>93.5% (15 min agitation)<br>98.2% (5 min no agitation)<br>98.8% (15 min no agitation)		Deterioration from 14 to 21 days (20°C light/no light).<br><br>Stable for 28 days (4°C no light).<br><br>Rapid deterioration within 24 hours (37 °C no light).<br><br>No statistical impact on (AC vs non-AC)<br><br>HGA:<br>Poor stability (20°C light/no light).<br><br>Stability in non-acidified dried urine VAMS significantly > than non-acidified liquid urine (4 °C no light).<br><br>Poor stability (37°C no light).<br><br>No statistical impact on stability (AC vs non-AC).<br><br>**Additional notes:**	1.014 (slope)<br>0.279 (intercept)<br>0.985 (R^2)<br><br>Tyr:<br>0.965 (slope)<br>1.593 (intercept)<br>0.97 (R^2)<br><br>HGA<br>0.94 (slope)<br>62.127 (intercept)<br>0.983 (R^2)	

								No effect of AC on Mitra device tip Significant improvement in stability at lower temperatures except for PHE in acidified conditions.<br><br>**Increased stability observed at 4°C for approximately 28 days for all analytes (recommended transport and storage temperature).**		

Abbreviations: Phenylalanine (Phe), 2,3-13C215N-Phenylalanine (Phe-surrogate), whole blood (WB), room temperature (rt), Tyrosine (Tyr), seconds (s), hematocrit effects (HCT), homogentisic acid (HGA), volume (vol), and standard deviation (SD), AC(AC).
