# Supplementary material for: Volumetric Absorptive Microsampling in the Analysis of Endogenous Metabolites
Source: Metabolites. 2023 Sep 26;13(10):1038. doi: 10.3390/metabo13101038 (PMC10609074; doi:10.3390/metabo13101038)
Supplement: Supplementary file 1 [file metabolites-13-01038-s001.zip › Table S6.pdf]

Table S6. Analytical validation of the developed method for determination of amino acids and organic acids in VAMS

Analytes	Linearity (R^2)	Precision (CV%)	Recovery (%)	Stability	Additional data	Ref
**Amino acids:**<br>Ala<br>Arg<br>Asn<br>Asp<br>Betaine<br>Choline<br>Cr<br>Glu<br>Gln<br>His<br>IIe<br>Leu<br>Lys<br>Met<br>Orn<br>Phe<br>Pro<br>Sar<br>Ser<br>Taurine<br>Thr<br>Trp<br>Tyr<br>Val<br><br>**Organic acids**<br>2-HBA<br>2-OGA<br>3-HPA<br>CA<br>FUMA<br>GSH	r^2 > 0.99	1.6% CV - 8.3% CV<br>(10, 25, 50, and 100 % concentration)	**Amino acids:**<br>Ala: 93%<br>Arg: 47%<br>Asn: 89%<br>Asp: 87%<br>Betaine: 97%<br>Choline: 92%<br>Cr: 92%<br>Glu: 93%<br>Gln: 85%<br>His: 89%<br>IIe: 95%<br>Leu: 85%<br>Lys: 68%<br>Met: 92%<br>Orn: 86%<br>Phe: 87%<br>Pro: 97%<br>Sar: 90%<br>Ser: 93%<br>Taurine: 96%<br>Thr: 91%<br>Trp: 78%<br>Tyr: 90%<br>Val: 101%<br>(10, 25, 50, and 100 % concentration)<br>**Organic acids**<br>2-HBA: 97%<br>2-OGA: 5%<br>3-HPA: 74%<br>CA: 60%<br>FUMA: 84%<br>GSH: 89%<br>Glyoxylic acid: 23%<br>LAC: 93%	**Stability of metabolites on dried whole blood samples on VAMS tips after storage at ambient temperatures**<br><br>Amino acids and organic acids<br><br>4 days:<br>88% - 130% (+/- < 13 SD)<br><br>7 days:<br>69% - 133% (+/- 12 SD)<br><br>15 days:<br>68% - 138% (+/- 10 SD)<br><br>No signal difference in VAMS dried and stored at room temperature for 0.5 to 26 hours. Difference in signal in venous blood stored at rt.	**Selectivity**<br>Isobaric compounds and metabolites with equal nominal mass were separated. No interferences at selected transitions.<br><br>**Note**<br>LOQ, accuracy, matrix effects, and HCT effects were not presented.	[38]

Glyoxylic acid<br>LAC<br>Malic acid<br>Pyr<br>SA<br>UA				Malic acid: 42%<br>Pyr: 80%<br>SA: 69%<br>UA: 60%<br>(10, 25, 50, and 100 % concentration)			
MMA<br>SA<br>KMV<br>KIC<br>ILE<br>Leu<br>Phe<br>Tyr<br>Valine	10.0-10,000 ng/ml	Intra-Day<br>LLOQ: < 12.5%<br>LQC: <8.9%<br>MQC: <5.3%<br>HQC: <5.5%	Inter-Day<br>LLOQ: < 5.3%<br>LQC: <4.5%<br>MQC: <4.3%<br>HQC: <4.2%	**At 30 ng/ml**<br>MMA: 91.8 %<br>SA: 106.67 %<br>KMV: 97.16 %<br>KIC: 77.51 %<br>ILE: 101.34 %<br>Leu: 103.14 %<br>Phe: 105.75 %<br>Tyr: 108.09 %<br>Valine: 103.51 %<br><br>**At 7500 ng/ml**<br>MMA: 63.4%<br>SA: 103.07%<br>KMV: 72.77%<br>KIC: 62.87%<br>ILE: 90.59 %<br>Leu: 93.02%<br>Phe: 88.45%<br>Tyr: 90.73%<br>Valine: 94.51%	Seven days in ambient conditions and at -80°C.<br><br>Three freeze/thaw cycles.	**Accuracy**<br>Acceptable (< than 15%)	[39]

Abbreviations: Phenylalanine (Phe), room temperature (rt), Tyrosine (Tyr), seconds (s), hematocrit effects (HCT), alanine (Ala), arginine (Arg), asparagine (Asn), aspartic acid (Asp), betaine (BL), choline, creatine (Cr), glutamic acid (Glu), glutamine (Gln), histidine (His), isoleucine (IIe), leucine (Leu), lysine (Lys), methionine (Met), ornithine (Orn), Phenylalanine (Phe), proline (Pro), sarcosine (Sar), serine (Ser), taurine, threonine (Thr), tryptophan (Trp), Tyrosine (Tyr), valine (Val), 2-hydroxybutanoic acid (2-HBA), 2-oxo-glutaric acid* (2-OGA), 3-hydroxypropionic acid (3-HPA), citric acid (CA), fumaric acid (FUMA), glutathione (GSH), glyoxylic acid, lactic acid (LAC), malic acid, pyruvic acid (Pyr), succinic acid (SA), uric acid (UA), methylmalonic acid (MMA), 3-methyl-2-oxovaleric acid sodium salt (KMV), sodium 4-methyl 2-oxo valerate (KIC).
