# Supplementary material for: Volumetric Absorptive Microsampling in the Analysis of Endogenous Metabolites
Source: Metabolites. 2023 Sep 26;13(10):1038. doi: 10.3390/metabo13101038 (PMC10609074; doi:10.3390/metabo13101038)
Supplement: Supplementary file 1 [file metabolites-13-01038-s001.zip › Table S7.pdf]

Table S7. Analytical validation of the developed method for tryptophan pathway metabolites determination in VAMS

Analytes	Linearity Range (ng/mL); (R^2)		LOQ (ng/mL)	Intra-Day Precision RSD%)	Inter-Day Precision (RSD%)	Accuracy (% bias)	Matrix Effects (%)	Recovery (%)	Hematocrit effects	Stability	Additional data	Ref
TRP	25-10,000	0.9988	25	4.5% - 7.9% RSD (low, medium, and high concentrations)	4.7% - 9.5% RSD (low, medium, and high concentrations)	90% - 107% (low, medium, and high concentrations)	87% - 96% (low, medium, and high concentrations)	86% - 96% (low, medium, and high concentrations)	–	86% - 95% (VAMS stored in a clamshell, in a bag with desiccant, protected from light at room temperature for 30 days).<br><br>> 85% (extracted samples stored in the autosampler for 24 hours at room temperature).<br><br>> 85% (extracted samples stored on the benchtop and exposed to all lab conditions for 24 hours).<br><br>87% - 91% for acidic compounds; 90% - 94% for other compounds (VAMS stored in a clamshell, in a bag with desiccant, protected from light at room temperature for > 30 days).	**Selectivity** No interference peaks at target retention time.<br><br>**Carryover** No signal in the blank was higher than background noise.	[10]
NFK	0.3-100	0.9991	0.3									
KYN	5-1000	0.9989	5									
ATA	0.2-100	0.9993	0.2									
KYNA	0.2-100	0.9992	0.2									
3-HKYN	0.2-100	0.9993	0.2									
XA	0.2-100	0.9994	0.2									
3-HAA	0.2-100	0.9992	0.2									
QUIN	1-500	0.9991	1									
PIC	1-500	0.999	1									
2-AMA	0.1-50	0.9995	0.1									
5-HTP	0.1-50	0.9994	0.1									
5-HT	1-500	0.9991	1									
5-HIAA	0.1-50	0.9996	0.1									
MEL	0.2-100	0.9994	0.2									
IND	0.1-50	0.9994	0.1									
TA	0.1-50	0.9992	0.1									
IDE	0.1-50	0.9993	0.1									
IPA	1-500	0.9989	1									
ILA	1-500	0.9991	1									
IAA	1-500	0.9991	1									
SK	0.1-50	0.9994	0.1									
IALD	0.2-100	0.9992	0.2									
IA	0.2-100	0.9993	0.2									

Abbreviations: L-tryptophan (TRP), N-formylkynurenine (NFK), kynurenine (KYN), kynurenic acid (KYNA), 3-hydroxykynurenine (3-HKYN), anthranilic acid (ATA), kynurenine aminotransferase (KAT), serotonin (5-HT), melatonin (MEL), xanthurenic acid (XA), 3-hydroxyanthranilic acid (3-HAA), quinolinic acid (QUIN), picolinic acid (PIC), 2-aminomuconic acid (2-AMA), 5-hydroxytryptophan (5-HTP), 5-hydroxyindole acetic acid (5-HIAA), melatonin (MEL), indole (IND), tryptamine (TA), 3- indoleethanol (IDE), 3-indolepropionic acid (IPA), indolelactic acid (ILA), 3-indoleacetic acid (IAA), skatole (SK), 3-indolealdehyde (IALD), and indoleacrylic acid (IA), relative standard deviation (RSD).
